# Unraveling the Rat Intestine, Spleen and Liver Genome-Wide Transcriptome after the Oral Administration of Lavender Oil by a Two-Color Dye-Swap DNA Microarray Approach

**DOI:** 10.1371/journal.pone.0129951

**Published:** 2015-07-10

**Authors:** Hiroko Kubo, Junko Shibato, Tomomi Saito, Tetsuo Ogawa, Randeep Rakwal, Seiji Shioda

**Affiliations:** 1 Department of Anatomy I, Showa University School of Medicine, Shinagawa, Tokyo, Japan; 2 Oriental Aromatherapy College, Katsushika, Tokyo, Japan; 3 Global Research Center for Innovative Life Science, Peptide Drug Innovation, School of Pharmacy and Pharmaceutical Sciences, Hoshi University, Shinagawa, Tokyo, Japan; 4 Laboratory of Exercise Biochemistry and Neuroendocrinology, Institute of Health and Sport Sciences, University of Tsukuba, Tsukuba, Ibaraki, Japan; 5 Department of Physiology, Saitama Medical University, Iruma-gun, Saitama, Japan; 6 Organization for Educational Initiatives, University of Tsukuba, Tsukuba, Ibaraki, Japan; 7 Faculty of Health and Sport Sciences & Tsukuba International Academy for Sport Studies (TIAS), University of Tsukuba, Tsukuba, Ibaraki, Japan; University of Jaén, SPAIN

## Abstract

The use of lavender oil (LO) – a commonly, used oil in aromatherapy, with well-defined volatile components linalool and linalyl acetate – in non-traditional medicine is increasing globally. To understand and demonstrate the potential positive effects of LO on the body, we have established an animal model in this current study, investigating the orally administered LO effects genome wide in the rat small intestine, spleen, and liver. The rats were administered LO at 5 mg/kg (usual therapeutic dose in humans) followed by the screening of differentially expressed genes in the tissues, using a 4×44-K whole-genome rat chip (Agilent microarray platform; Agilent Technologies, Palo Alto, CA, USA) in conjunction with a dye-swap approach, a novelty of this study. Fourteen days after LO treatment and compared with a control group (sham), a total of 156 and 154 up (≧ 1.5-fold)- and down (≦ 0.75-fold)-regulated genes, 174 and 66 up- (≧ 1.5-fold)- and down (≦ 0.75-fold)-regulated genes, and 222 and 322 up- (≧ 1.5-fold)- and down (≦ 0.75-fold)-regulated genes showed differential expression at the mRNA level in the small intestine, spleen and liver, respectively. The reverse transcription-polymerase chain reaction (RT-PCR) validation of highly up- and down-regulated genes confirmed the regulation of the *Papd4*, *Lrp1b*, *Alb*, *Cyr61*, *Cyp2c*, and *Cxcl1* genes by LO as examples in these tissues. Using bioinformatics, including Ingenuity Pathway Analysis (IPA), differentially expressed genes were functionally categorized by their Gene Ontology (GO) and biological function and network analysis, revealing their diverse functions and potential roles in LO-mediated effects in rat. Further IPA analysis in particular unraveled the presence of novel genes, such as *Papd4*, *Or8k5*, *Gprc5b*, *Taar5*, *Trpc6*, *Pld2* and *Onecut3* (up-regulated top molecules) and *Tnf*, *Slc45a4*, *Slc25a23* and *Samt4* (down-regulated top molecules), to be influenced by LO treatment in the small intestine, spleen and liver, respectively. These results are the first such inventory of genes that are affected by lavender essential oil (LO) in an animal model, forming the basis for further in-depth bioinformatics and functional analyses and investigation.

## Introduction

Aromatherapy has been defined or is referred to as “*the use of concentrated essential oils that are extracted from herbs*, *flowers and other plant parts to treat various diseases*” (quoted from a systematic first review by [1,2]). Historically claimed to be an ancient practice among early civilizations, it was only in 1936 that term aromatherapy was used by the French chemist Gattefosse [3,4]. Although essential oils (which are usually distilled from medicinal plants or herbs) have been traditionally used in Japan [5] and are generally known for providing vibrant health and beauty, the official beginning of associations dealing with aromatherapy started in 1996 (Aroma Environment Association of Japan, http://www.aromakankyo.or.jp/english/aeaj.htmlJ; Japanese Society of Aromatherapy (JSA), http://aroma-jsa.jp/). For further reading on medical and clinical aromatherapy, readers are referred to [6] and [7].

Among the Japanese people, aromatherapy is widely used or applied and in numerous ways [8]; the main application methods of essential oils include the inhalation of the volatile components (e.g., menthol, limone, and linaol vapors) and the percutaneous absorption via the application of diluted oil to the skin (e.g., sweet almond, Jojoba, grape seed oils). On the other hand, the oral administration of essential oils (e.g., lavender oil; hereafter abbreviated as LO) is traditionally performed in some European countries [9] but is not popular in Japan. For example, in France, Belgium, and Germany, LO is used as a form of herbal or natural medicine. Furthermore, a German pharmaceutical company produces an encapsulated form of LO preparation, Silexan (W. Spitzner Arzmeimittelfabrik GmbH, Ettlingen, Germany). Recent animal and clinical studies have generally shown beneficial effects of Silexan [10]. The reasons behind the non-use of LO in Japan might be cultural or scientific; the authors believe that there is no concrete evidence or studies for that matter regarding the safety aspects of ingesting essential oils (for example LO).

Lavender oil, which is distilled from the species *Lavandula angustifolia* (Labiatae), is the source for the commonly used oil in aromatherapy [11–13] and the subject of our current investigation. The reason for the use of LO is first because LO is one of the most popular essential oils that are used in aromatherapy and whose use in non-traditional medicine is increasing worldwide. Second, LO has the well-defined volatile components linalool and linalyl acetate in almost equal amounts [14], the effect of which can be examined via the administration of LO as oil. Although there some studies have investigated the effects of LO using the commercial formulation Silexan [15], there is no study using a pure (100%) LO. The main novelty of this study is however the use of an omics-based approach, namely transcriptomics by whole genome DNA microarray [16] analysis of a rat model to which LO was orally administered LO. To best of our knowledge, there is no such study employing a genome-wide approach.

**Fig 1:**
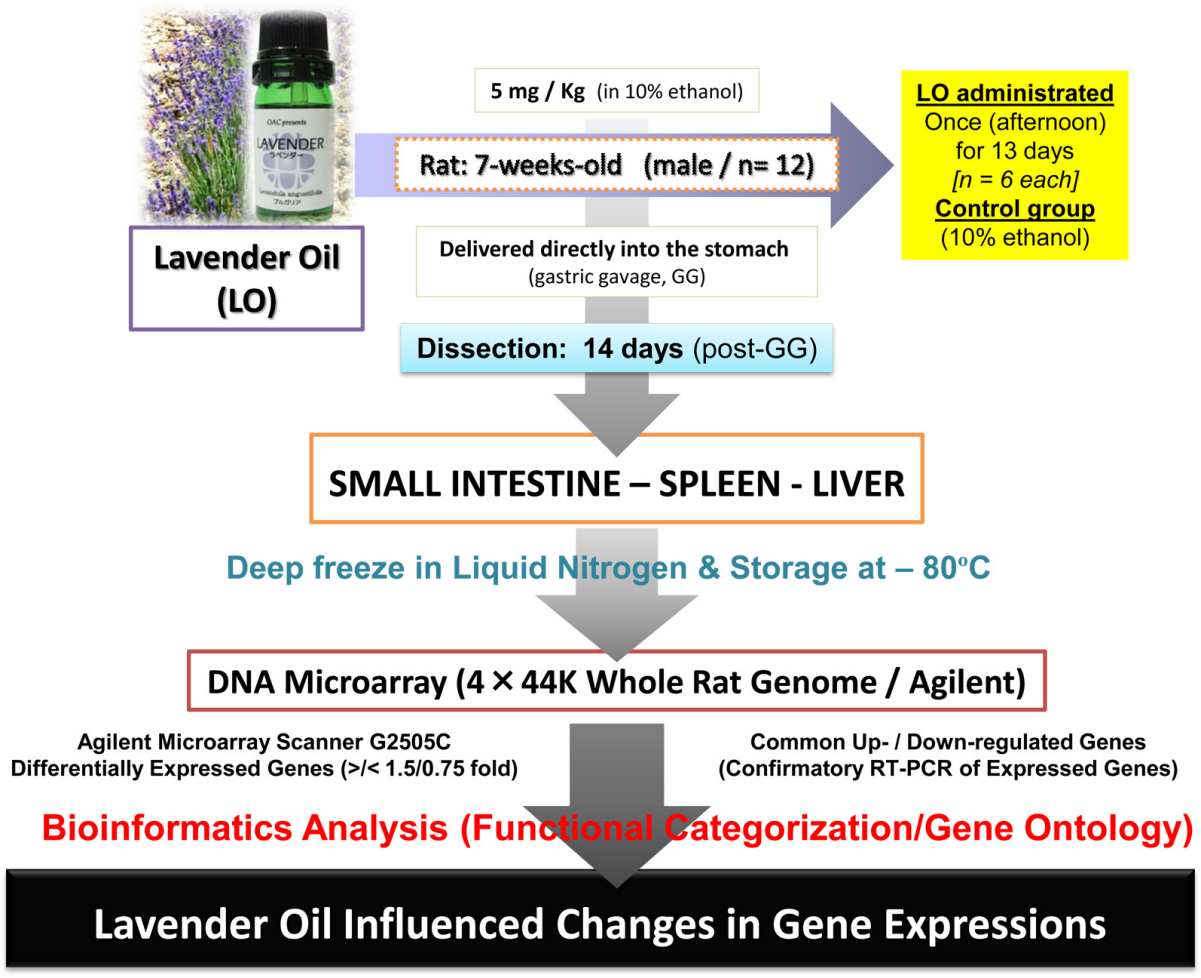
The experimental strategy to screen for genes that are influenced by lavender oil (LO). Rats (7 weeks old) were given lavender oil (LO) orally once each day for 13 days, and on the 14^th^ day, the small intestine, spleen and liver were dissected out and deep frozen in liquid nitrogen. DNA microarray analysis was performed using a rat whole-genome DNA microarray chip (G4131F, Agilent), and the differentially expressed genes were identified in the LO-treated tissues followed by bioinformatics analyses. For further details, see the Materials and Methods section.

To unravel the LO-influenced genes in the rat model, we first carried out a pilot study to confirm the incorporation of two major LO components, linalool and linalyl acetate, in the blood post-oral administration. The pilot study quantified linalool and linalyl acetate in the rat plasma after oral administration of LO (**S1 Fig)**. The venous blood from the heart and the portal vein was collected and the presence or absence of these two components was determined using a gas chromatography-mass spectrometry (GC-MS) technique. GC-MS analysis revealed the presence of linalool and linalyl acetate in the portal vein and this was quantified in a time-dependent manner (**S1 Fig)**. However, these two components could not be detected from the heart (data not shown). As both linalool and linalyl acetate were found in the blood (portal vein), LO was indeed incorporated into the body of the rat, and which provided confidence to take the experiment to the next step toward investigating effects of orally administered LO in the rat model. For the DNA microarray experiments, the rats were orally administered LO at 5 mg/kg (the usual therapeutic dose in humans) followed by the screening of differentially expressed genes in the small intestine, spleen, and liver, using a 4×44-K whole genome rat chip (Agilent) and the dye-swap approach [17–22]. The experimental strategy is illustrated in **Fig 1**. It was hypothesized that LO administration at a low-dose would induce changes in the gene expression profiles starting with the small intestine, where the oil is perceived and ingested, and probably acting as a site for first pass metabolism, followed by LO (linalool and linalyl acetate) transport through the blood to the liver (and spleen), where further metabolism takes place [23].

As expected, our results indicate that LO influences the expression of numerous genes in these tissues/organs. These results are i) presented as a resource for the scientific community as a first such inventory of genes that are affected by LO in a rat model and ii) discussed in the context of the use of LO for human health and therapeutic properties. Moreover, using bioinformatics approaches, these genes have been functionally categorized by their Gene Ontology (GO) and are presented and briefly discussed in line with available literature with an aim of correlating some of the changed gene expressions with LO affects.

## Materials and Methods

### Animals and husbandry and ethics statement

Fischer (F334) rats were purchased from Japan SCL (Hamamatsu, Japan). Twelve male rats (7 weeks old; ~142.8 to 167.5-g body weight) were housed at the Animal Institution in Showa University. The rats were maintained in individual cages in isolated animal rooms with controlled temperature and relative humidity with a 12-h light: 12-h dark schedule (lights were turned on at 08:00 AM) and had access to laboratory chow and tap water ad libitum. All of the animal experiments began after a period of acclimation of at least one week. All of the animal care and experimental procedures were approved by the Institutional Animal Care and Use Committee of Showa University (approval number M6031).

### Administration of lavender oil, dissection and sampling of tissues for molecular analysis

Lavender oil (LO; *L*. *angustifolia*, Biofloral, France) was administered to rats (n = 6 per each group) directly into the stomach at a dose of 0 (10% ethanol; diluent: control group) or 5 mg/kg (in 10% ethanol: LO-administered group) once a day in the afternoon (11:25–13:50) for 13 days. Fourteen days after treatment, the rats were dissected under ether anesthesia. The small intestine was sampled approximately 20 cm from the duodenum and washed by saline (0.9% NaCl). The whole spleen was dissected out. Two blocks of 8 mm^2^ were sampled from the whole liver. All of the dissected tissues were immediately immersed in liquid nitrogen (N_2_) and stored at -80°C in a deep freezer.

### Total RNA extraction, cDNA synthesis and reverse transcription-polymerase chain reaction (RT-PCR)

The deep-frozen tissues were transferred to a pre-chilled (in liquid nitrogen) mortar and ground with a pestle to a very fine powder with liquid nitrogen. Sample powders (small intestine, spleen, and liver) were stored at -80°C until used for RNA extraction. The total RNA was extracted from ~60-mg sample powder using the Qiagen RNeasy Mini Kit (Qiagen, Maryland, MD, USA) [17–22]. The protocol is schematically described for reference in **S2 Fig**. To verify the quality of this RNA, the yield and purity were determined spectrophotometrically with NanoDrop (Thermo Scientific, Wilmington, DE, USA) and confirmed using formaldehyde-agarose gel electrophoresis (data not shown). The cDNA synthesis and quality check by RT-PCR were also previously described [17–22]. The synthesized cDNA quality was checked using RT-PCR to confirm the expression of the house-keeping gene glyceraldehyde 3-phosphate dehydrogenase (*Gapdh*) as a positive control [24] (data not shown). The gene-specific primer design is presented in **Table 1**.

**Table 1:** Gene-specific primers that were designed in-house and used for the RT-PCR analysis of gene expression.

	Forward Primer		Reverse Primer			
Accession (Gene)	Primer name	Nucleotide sequence (5'-3')	Primer name	Nucleotide sequence (5'-3')	Product size (bp)	Gene Name
NM_017008	SR001	cctgtgacttcaacagcaactc	SR002	ggcctctctcttgctctcagta	213	*Gapdh*
NM_001107843	SR045	aaaaatttggtcatggctcagt	SR046	caccttcctcctgaataactgg	305	*Lrp1b*
NM_001008372	SR049	ccccatgtaatgttcctcctta	SR050	tcttttcagggtagcagctctc	338	*Papd4*
NM_134326	SR041	caccaagtgttgtacccttcct	SR042	tgggagtgtgcagatatcagag	250	*Alb*
AB015877	SR033	gtccttgtggacaaccagtgta	SR034	cctttagtccctgaacttgtgg	341	*CYR61*
NM_031572	SR031	atggtttgttgcatgaagtgac	SR032	aatggtggtcaggaacaaaaac	266	*Cyp2c12*
NM_030845	SR043	tccccaagtaatggagaaagaa	SR044	acacgatcccagactctcatct	349	*Cxcl1*

### Global gene expression analysis on a rat whole-genome DNA chip

**Fig 2:**
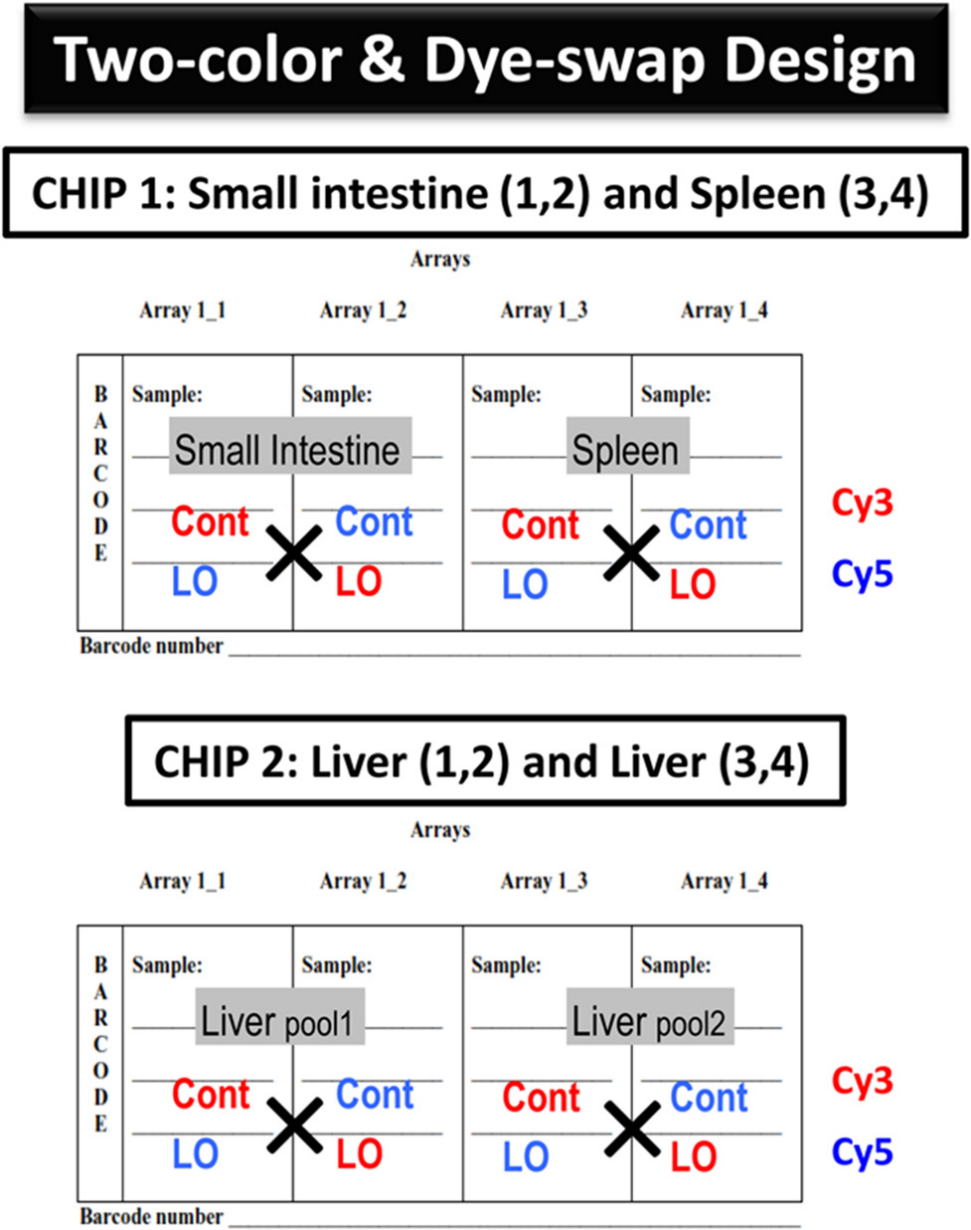
The two-color and dye-swap approach was used for genome-wide analysis. Two DNA chips were used for each sample condition, i.e., a dye-swap approach was used to label one set of RNAs by Cy 3 and the other by Cy5 for each control and LO sample, respectively. A 4×44-K format slide containing 4 chips is illustrated for the current study. For further details, see the Materials and Methods section.

The total RNA that was extracted from the small intestine, spleen, and liver tissues for each control and LO-administered sample was pooled in each group prior to DNA microarray analysis (Rat whole genome 4×44-K:G4131F; Agilent Technologies, Palo Alto, CA, USA). The reason for pooling the total RNA and not individual tissues (due to usually larger variability at the tissue level than at the RNA level) for estimating the gene expression is two-fold. First, pooling of the samples (total RNA) resulted in the use of lesser number of chips that was an economical/cost-effective option, in this study due to large number of tissues to be analyzed, for the highly expensive microarray analysis [25]. Second, as there will be some level of biological variability amongst the samples, we pooled the extracted total RNA to average out the possible differences in expression of genes. A study on statistical implications of pooling RNA samples also suggested that gene expression estimates are more precise in the pooled samples, assuming mRNA abundances in individual samples averages out when pooled [26]. To note, a recent paper has taken forward the concept of pooling mRNA samples in microarray experiments further by suggesting the use of a smart pooling approach [27]. The total RNA (200 ng) was labeled with either Cy3 or Cy5 dye using an Agilent Low-RNA-Input Fluorescent Linear Amplification Kit (Agilent Technologies, Santa Clara, CA, USA). The fluorescently labeled targets of the control as well as the treated samples were hybridized to the same microarray slide with 60-mer probes. A flip-labeling (dye-swap or reverse labeling with Cy3 and Cy5 dyes) procedure was followed to nullify the dye bias that was associated with the unequal incorporation of the two Cy dyes into cDNA (**Fig 2**). Briefly, to select differentially expressed genes by the dye-swap approach, we considered genes that were up-regulated in chip 1 (Cy3/Cy5 label for control and LO-administered samples, respectively) but down-regulated in chip 2 (Cy3/Cy5 label for LO-administered and control samples, respectively) for small intestine tissue. Similarly, the same selection criteria were applied for chips 3 and 4 (spleen tissue), for chips 5 and 6 (liver tissue pool 1) and for chips 7 and 8 (liver tissues pool 2). The use of a dye-swap approach provides more-stringent selection conditions for changed gene expression profiling than the use of a simple single/two-color approach [28]. The hybridization and wash processes were performed according to the manufacturer’s instructions, and the hybridized microarrays were scanned using an Agilent Microarray scanner G2505C. To detect significantly differentially expressed genes between the control and treated samples, each slide image was processed using Agilent Feature Extraction software (version 11.0.1.1). This program measures the Cy3 and Cy5 signal intensities of whole probes. Dye bias tends to be signal-intensity-dependent; therefore, the software selected probes using a set-by-rank consistency filter for dye normalization. Said normalization was performed by LOWESS (locally weighted linear regression), which calculates the log ratio of dye-normalized Cy3- and Cy5 signals and the final error of the log ratio. Statistical significance of the log ratio values obtained for individual microarray probes was calculated using the “most conservative error model” default function of the Feature Extraction software, by which both a propagated and a universal error model are evaluated, and the higher (more conservative estimate of error) p-value of two error models was reported (Agilent Technologies [28]). In this analyses, the threshold of significant differentially expressed genes was <0.01 (for the confidence that the feature was not differentially expressed). Additionally, the erroneous data generated due to artifacts were eliminated before data analysis by the software.

To re-check some of the microarray data for semi-quantitative RT-PCR, an accepted methodology to validate the microarray results in biological sciences, as detailed above, was performed on selected genes using gene-specific primers (**Table 1**). Each gene expression analysis was performed at least twice as independent PCR reactions and electrophoresis on a gel for each cDNA (n = 6). The average mRNA abundance for each gene (control and LO) is presented.

### Functional categorization

Pathway and disease state-focused gene classification of the differentially expressed genes in the small intestine, spleen, and liver were classified based on the available categories of more than 100 biological pathways or specific disease states in the SABiosciences PCR array list (Qiagen; www.sabiosciences.com) for *Rattus norvegicus*.

### Ingenuity Pathway Analysis (IPA)

The biological function and network analyses were performed using IPA (Ingenuity Systems, www.ingenuity.com). Following our original submission, and during the revision, a new analysis was performed using the IPA tool (Content version: 23814503, Release Date: 2015-03-22, Qiagen). The dataset from the microarray (LO treatment in the small intestine, spleen, and liver), which includes the differentially expressed (≧/≦ 1.5/0.75-fold compared with control) genes and their corresponding fold change values, was uploaded as an Excel spread sheet into the IPA tool. To create gene networks, the genes were overlaid onto a global molecular network that was developed from information that was contained in the ingenuity knowledge base. The functional analysis identified the biological functions that were most significant to the dataset (p-value < 0.05) according to a right-tailed Fisher’s exact test.

### Access to gene array data

The outputs of the microarray analysis that were used in this study are available under the number series GSE 63640 (whole genome) at the NCBI Gene Expression Omnibus (GEO) public functional genomics data repository (http://www.ncbi.nlm.nih.gov/geo/query/acc.cgi?acc=GSE63640).

## Results

### Experimental strategy, dose and timing of lavender oil administration and tissues sampling

The main compounds of LO are linalool (31.85%), linalyl acetate (31.00%), β-ocimene (8.85%), and 1,8-cineole (4.4%) as described in the chemical datasheet by Biofloral. As neither linalool nor linalyl acetate were present in the heart (venous blood) but were detected from the portal vein (**S1 Fig**), we considered the first-pass effects in the liver after the oral administration of lavender essential oil (LO). To note, a study in 1990 demonstrated the presence of LO fragrance compounds in blood samples of rat [29]. The presence of these two components in the blood has also previously been shown in human subject’s blood samples after transdermal application of LO in a 1992 study [30] and following oral administration of LO preparation Silexan in 2010 and 2013 studies [31,32]. There are no other reports to our knowledge in animal models, and which also raises the question of the metabolism of these two major LO components. According to the PubChem compound database, hydrolysis of linalyl acetate occurs in the gastric fluids and the reaction products are linalool and acetic acid; therefore it has been mentioned that linalool would enter the systemic circulation [33]; for linalool, it is rapidly absorbed and thereafter metabolized [34]. We cannot explain this further without experimental evidences, but our results on the higher level of linalool may support this report. The path of LO is thus assumed from the small intestine and/or spleen via the portal venous system to the liver. As illustrated in **Fig 1**, the LO was administered orally to the rats (5 mg/kg once a day for 13 days), including the sham (control group, 10% ethanol), and 14 days after treatment, the intestine, spleen, and liver were dissected out to examine the differentially expressed genes.

The reason for selecting the time point and dose was based on a preliminary trial where LO was administrated to male Fischer rats at a dose of 0, 5 mg/kg (clinical dose), or 500 mg/kg (1/10 dose of the LD50). At one or six hours after the oral administration, we collected the liver and investigated into the genome-wide gene expression profiles on a rat whole genome 4×44-K DNA microarray chip (Kubo et al., unpublished data). For the 5 mg/kg LO dose, only 11 & 12 up (>1.5-fold)–and down (<0.75-fold)–regulated genes were found at one hour after administration, and 27 & 22 up (>1.5-fold)–and down (<0.75-fold)–regulated genes at six hours after LO administration. For the 500 mg/kg LO dose, 44 & 17 up (>1.5-fold)–and down (<0.75-fold)–regulated genes were found at one hour after administration, and 111 & 49 up (>1.5-fold)–and down (<0.75-fold)–regulated genes at six hours after LO administration. These low number of gene expression changes were insufficient to discuss about the influence of the oral administration of LO. In traditional way of use, usually 10 to 20 days of continuous daily administration of essential oils is used or recommended, followed by one-week interval prior to the next round. Keeping this time of administration and the preliminary data on 5 mg/kg over 500 mg/kg LO administration in mind, we focused on a two-week experiment using the low-dose (5 mg/kg), as described in Materials and Methods section, for determining the influence of LO on the body of rat.

### Total RNA extraction and confirmation of *Gapdh* gene expression by RT-PCR

To investigate global changes in gene expression in the small intestine, spleen, and liver, we first optimized a protocol for total RNA extraction, especially for the small intestine, for the first time in our research. The quantity and quality of the total RNA were confirmed, and this RNA was then used to synthesize cDNA. Prior to DNA microarray analysis, we examined of commonly used house-keeping gene *Gapdh* in the small intestine, spleen, and liver, allowing us to subsequently use this gene as a positive control rather than by simply loading or using internal controls [24]. This simple test of gene expression showed that the mRNAs for *Gapdh* were expressed almost uniformly across conditions; i.e., primarily to check its mRNA abundance in all samples and not as a mathematical adjustment or denominator for gene expression calculations. Recently, we showed similar expression levels (probe signal intensity) for the *Gapdh* gene with Cy3 and Cy5 labels in a microarray experiment on the ischemic brain [28]. Using the same analysis approach, we found that the Cy3 and Cy5 label associated expression for all the 10 *Gapdh* (probes) is similar across all probes plotted on the microarray chip (**S3 Fig)**. Results showed a good correlation between the Cy3 and Cy5 labels expression points across the 1.5-fold change ratio. Following this preliminary analysis of sample quantity and quality based on the total RNA check, we conducted a DNA microarray analysis with a dye-swap method (**Fig 2**) using the small intestine, spleen, and liver samples; pooled samples (total RNA) were used as detailed above in the Materials and Methods section. The reason for multiple checks as mentioned above is important in any gene expression analysis experiment and is especially true for a DNA microarray-based approach. The resulting data on gene expression changes can be easily misinterpreted if any of the above steps are not followed precisely.

### Global gene expression analysis by DNA microarray

**Fig 3:**
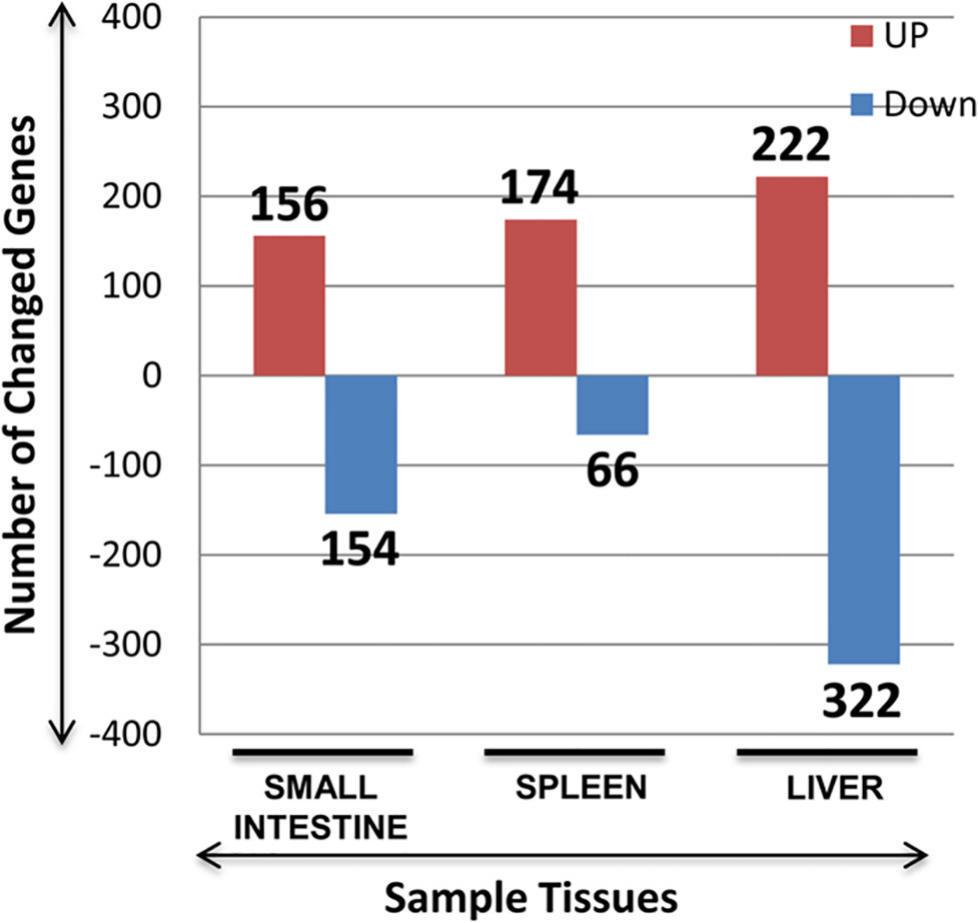
Differentially expressed genes in the small intestine, spleen, and liver. The histograms show the up (red)- and down (blue)-regulated gene (≧/≦ 1.5/0.75-fold) numbers in each examined tissue.

DNA microarray analysis revealed 156 and 154 up (>1.5-fold)- and down (<0.75-fold)-regulated genes in the small intestine, 174 and 66 up- and down-regulated genes in spleen, and 222 and 322 up- and down-regulated genes in liver (**Fig 3**). At a glance, up-regulated genes were predominant in small intestine and spleen, whereas the liver showed more down-regulated genes than up-regulated genes. The liver was also the organ with the largest numbers of gene expression changes. These genes are listed in **S1 Table** (small intestine), **S2 Table** (spleen), and **S3 Table** (liver). These results provide the first inventory of an essential oil (LO)-triggered gene expression in an animal (rat) model.

### Validatory RT-PCR analysis

**Fig 4:**
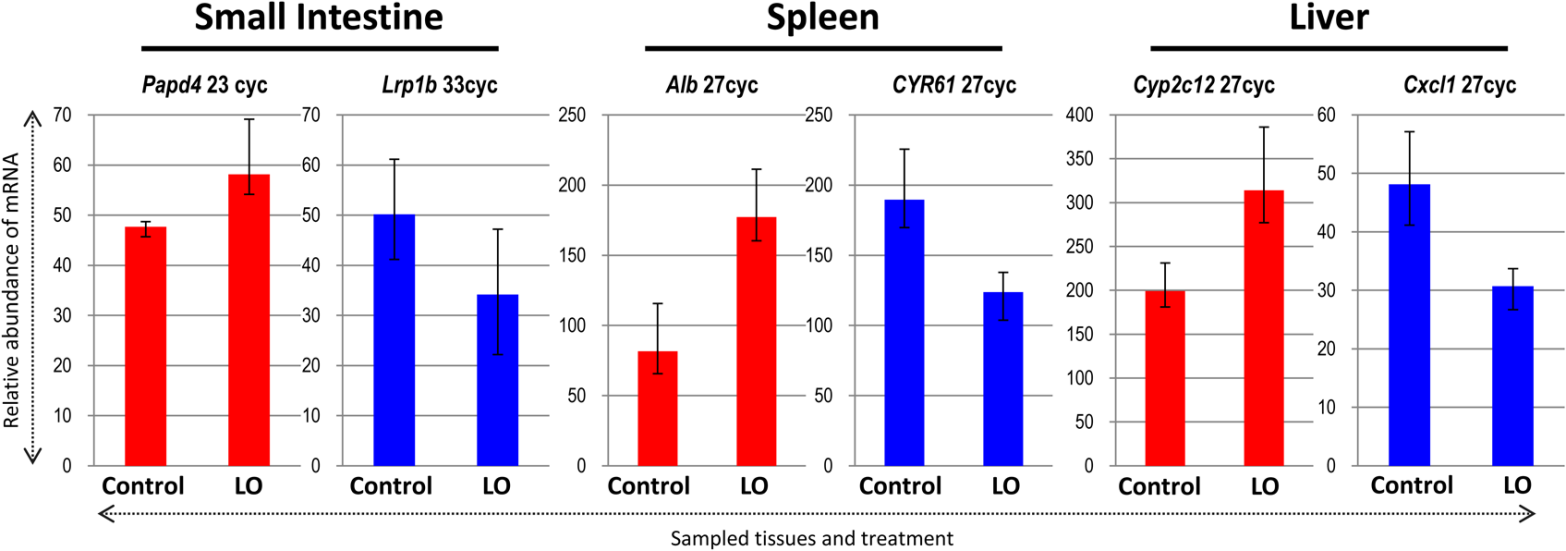
Validatory RT-PCR of selected differentially expressed up- and down-regulated genes in the small intestine, spleen, and liver. The graphs show the up (red)- and down (blue)-regulated genes (gene-specific primers are given in Table 1) in each tissue under the control and LO treatment. LO, lavender oil.

To confirm alterations in the gene expression as observed by DNA microarray, we selected one of each of the up- and down-regulated genes in the small intestine (*Papd4*, induced; *Lrp1b*, suppressed), spleen (*Alb*, induced; *Cyr61*, suppressed), and liver (*Cyp2c12*, induced; *Cxcl1*, suppressed); these genes and their specific primers are listed in **Table 1**. The DNA microarray data can be re-confirmed by RT-PCR using appropriate primer (gene-specific) design and using various cycles in order to measure the expression level under the most appropriate conditions (not at plateau) (**Fig 4**). Not only did we visually confirm the amplified bands on a gel (1.8% agar and Mupid-ex electrophoresis system, Advance, Tokyo, Japan), but also included a software/image analysis tool post-visualization using a ChemiDoc XRS+ (Bio-Rad Laboratories, Inc., Hercules, CA, USA) to quantify the area/intensity of the bands. The up- and down-regulation of the six genes were confirmed by RT-PCR, validating the obtained microarray experimental data.

### Functional categorization of differentially expressed genes by LO treatment

**Fig 5:**
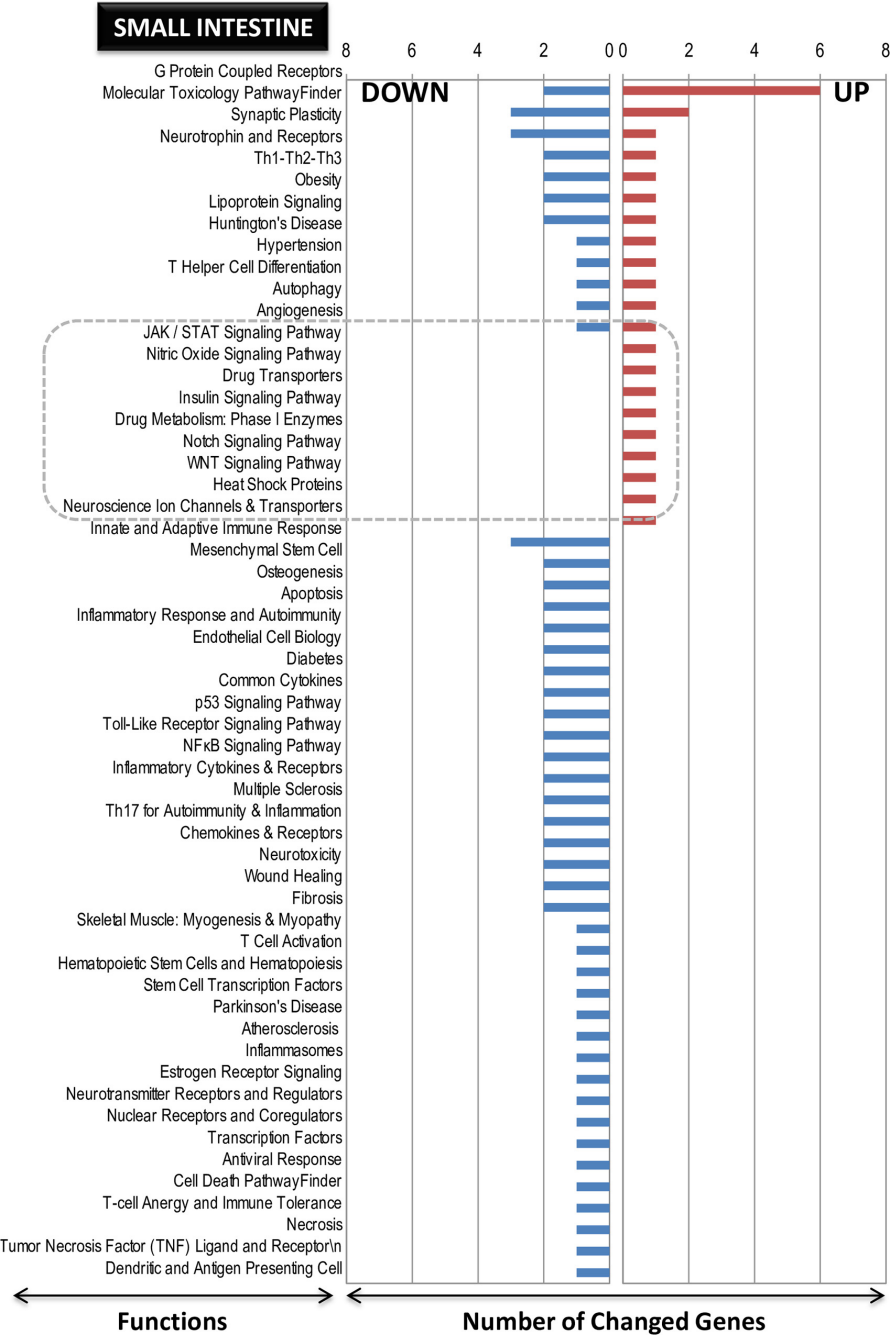
Pathway- and disease states-focused gene classification of genes in the small intestine of LO-treated rats. The genes (up-regulated–**A**; down-regulated–**B**) were classified based on the available categories of more than 100 biological pathways or specific disease states in the SABiosciences PCR array list (QIAGEN; www.sabiosciences.com) for *Rattus norvegicus*. The numbers in the Y-axis represent the number of genes in each category as indicated on the X-axis.

**Fig 6:**
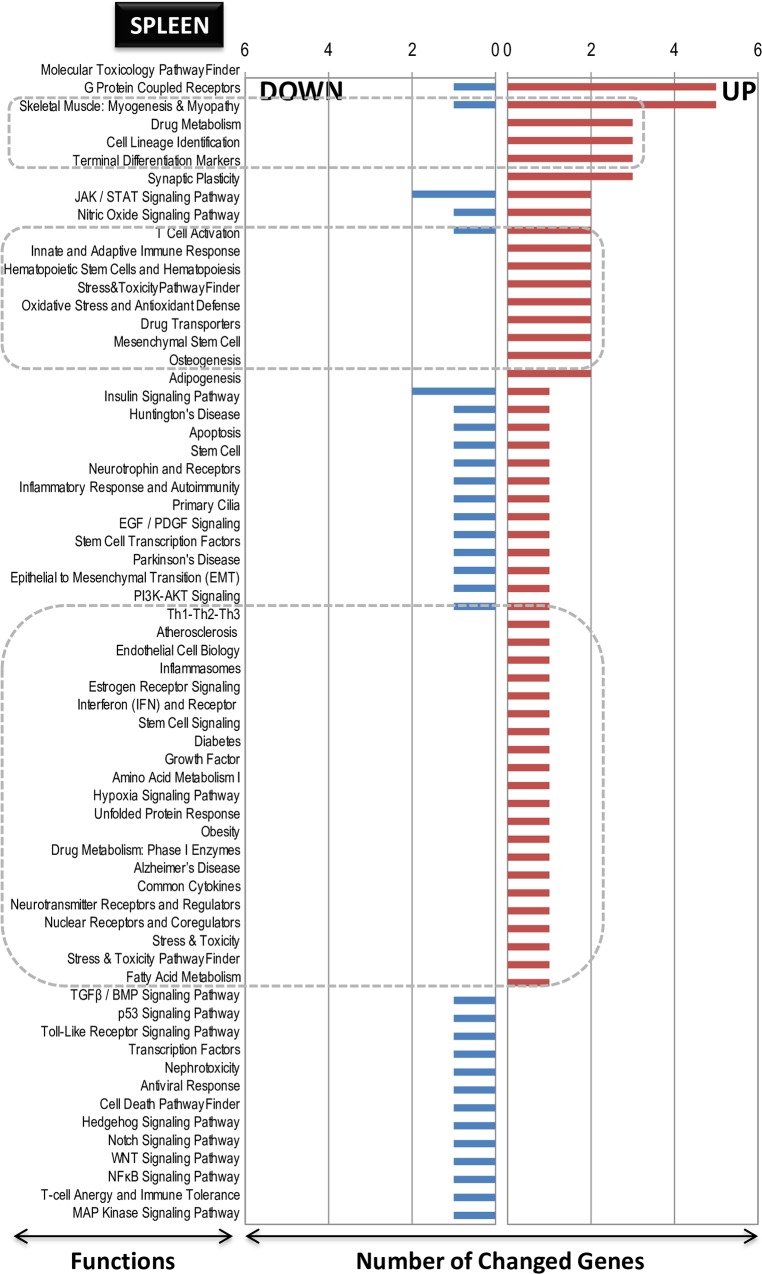
Pathway- and disease states-focused gene classification of genes in the spleen of LO-treated rats. The genes (up-regulated–**A**; down-regulated–**B**) were classified based on the available categories of more than 100 biological pathways or specific disease states in the SABiosciences PCR array list (QIAGEN; www.sabiosciences.com) for *Rattus norvegicus*. The numbers in the Y-axis represent the number of genes in each category as indicated on the X-axis.

**Fig 7:**
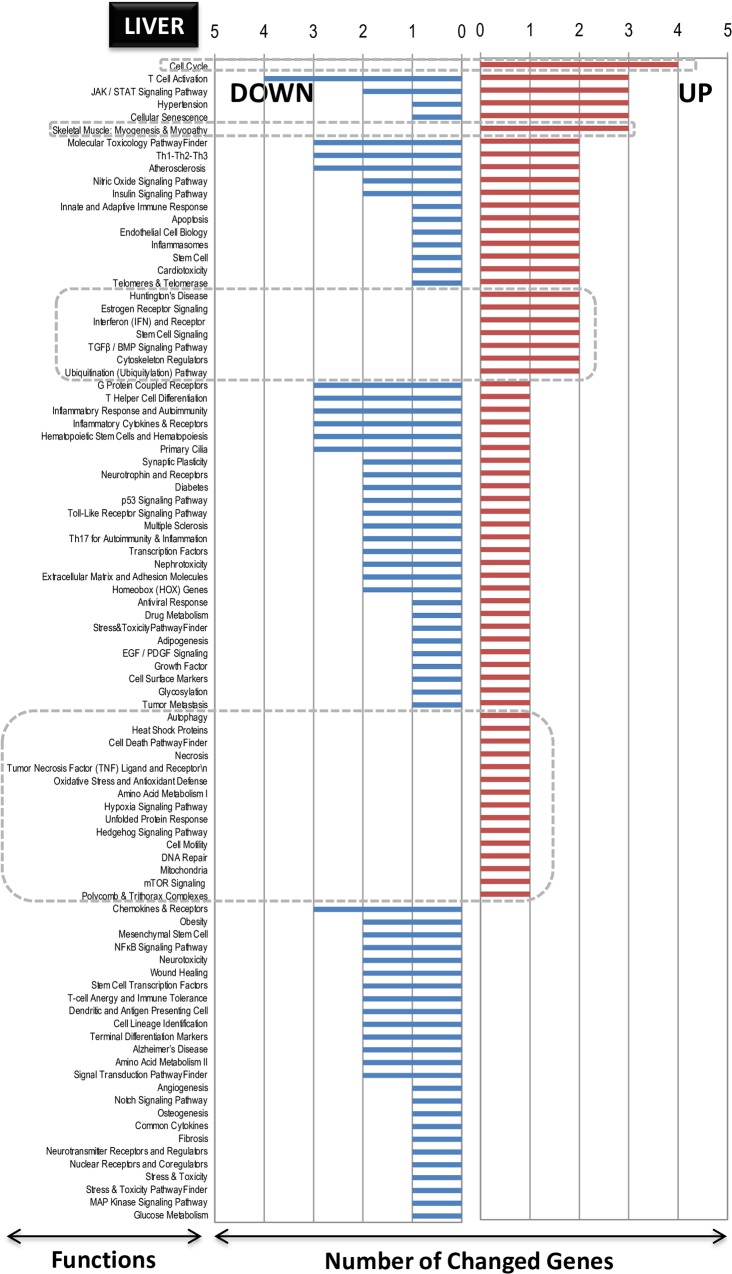
Pathway- and disease states-focused gene classification of genes in the liver of LO-treated rats. The genes (up-regulated–**A**; down-regulated–**B**) were classified based on the available categories of more than 100 biological pathways or specific disease states in the SABiosciences PCR array list (QIAGEN; www.sabiosciences.com) for *Rattus norvegicus*. The numbers in the Y-axis represent the number of genes in each category as indicated on the X-axis.

The functionally categorized genes post-analysis using the SABiosciences PCR array list (Qiagen) are presented graphically in **Fig 5** (small intestine), **Fig 6** (spleen) and **Fig 7** (liver). In **Figs 5** and **6** and **7** the numbers on the y-axis represent the number of genes in each category, which are indicated on the x-axis. The gene function and the genes description contained within each functional category are shown for small intestine (up-regulated, **S4 Table**; down-regulated, **S5 Table**), spleen (up-regulated, **S6 Table**; down-regulated, **S7 Table**), and liver (up-regulated, **S8 Table**; down-regulated, **S9 Table**). Some interesting facts could be ascertained using the known gene classifications. First, in the case of the small intestine, although up-regulated genes were more abundant (even by two genes), the functions were mainly down-regulated (**Fig 5**). In the case of the spleen, additional functions were correlated with additional up-regulated genes, as seen in **Fig 6**. In the liver, more down-regulated genes numbers also correlated with more functions (**Fig 7**). These functions were characterized by the presence of genes on both sides, up- and down-regulated. Interestingly and common to all the three tissues, among the up-regulated genes, there were pockets of functions that were specific to the induced genes only; these pockets are marked by boxed areas with broken lines (**Figs 5** and **6 and 7**), indicating that the LO specifically influenced some known genes and functions as up-regulation rather than down-regulation under the present analysis conditions of utilizing the well-annotated genes and functional categories as referenced from Qiagen (see Materials and Methods section above). In the small intestine, the *G protein-coupled receptors* gene function was the most highly up-regulated; in the spleen, the *molecular toxicology pathway finder* and *G protein coupled receptors* were the top-two highly up-regulated gene functions; and in the liver, the *cell cycle* was the highest up-regulated function. This regulation indicates diverse gene functions being influenced by LO ingestion in these three different tissues. Therefore, the first objective of identifying genes and their functions by DNA microarray in conjunction using an annotated gene function list was achieved.

**Fig 8:**
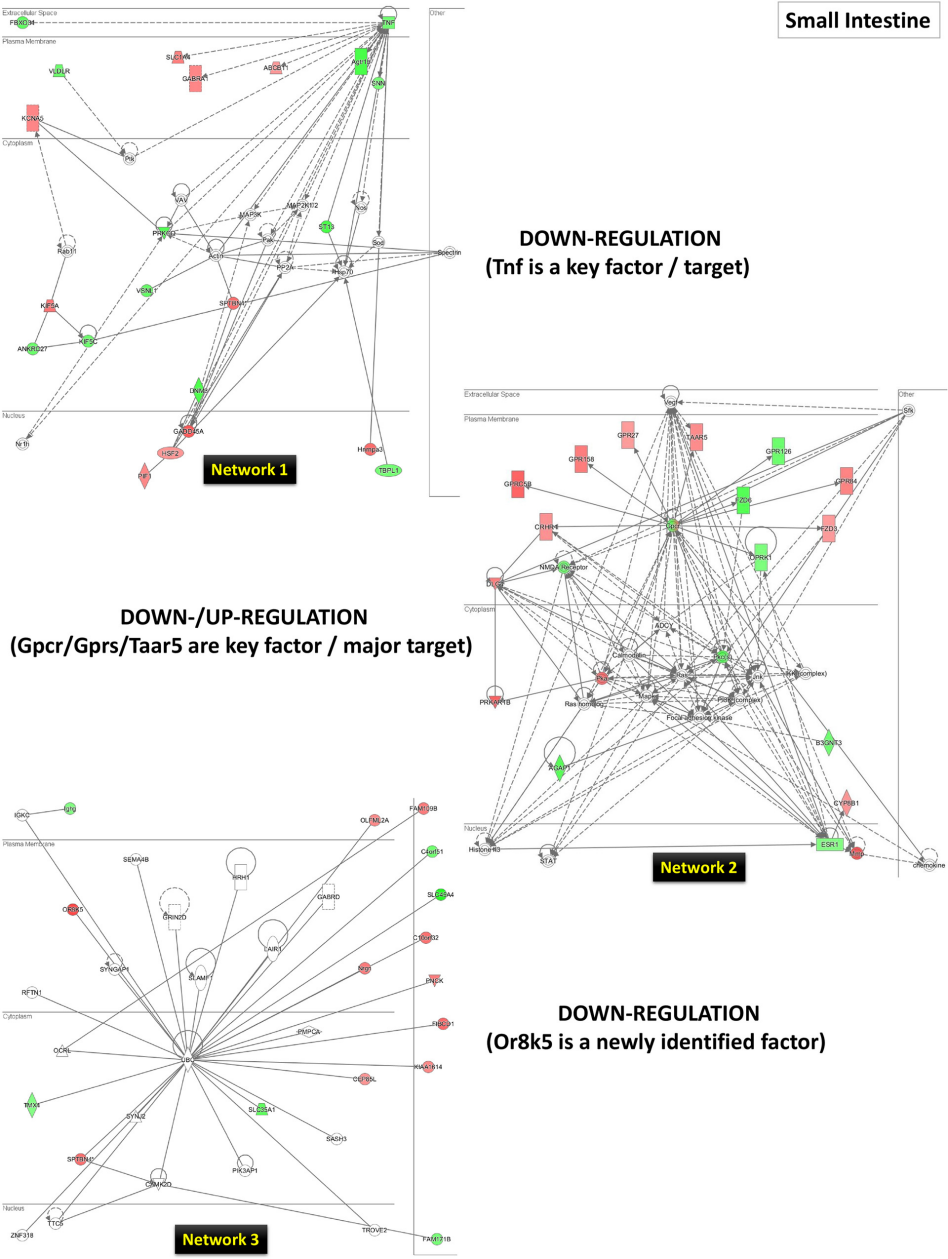
The top three networks (1, 2, and 3) for the small intestine by IPA analysis. Genes central to the pathway are placed aside each network in a box. The genes (up-regulated–red; down-regulated–green) are networked based on the evidence in the IPA. For further details, see the Materials and Methods section.

**Fig 9:**
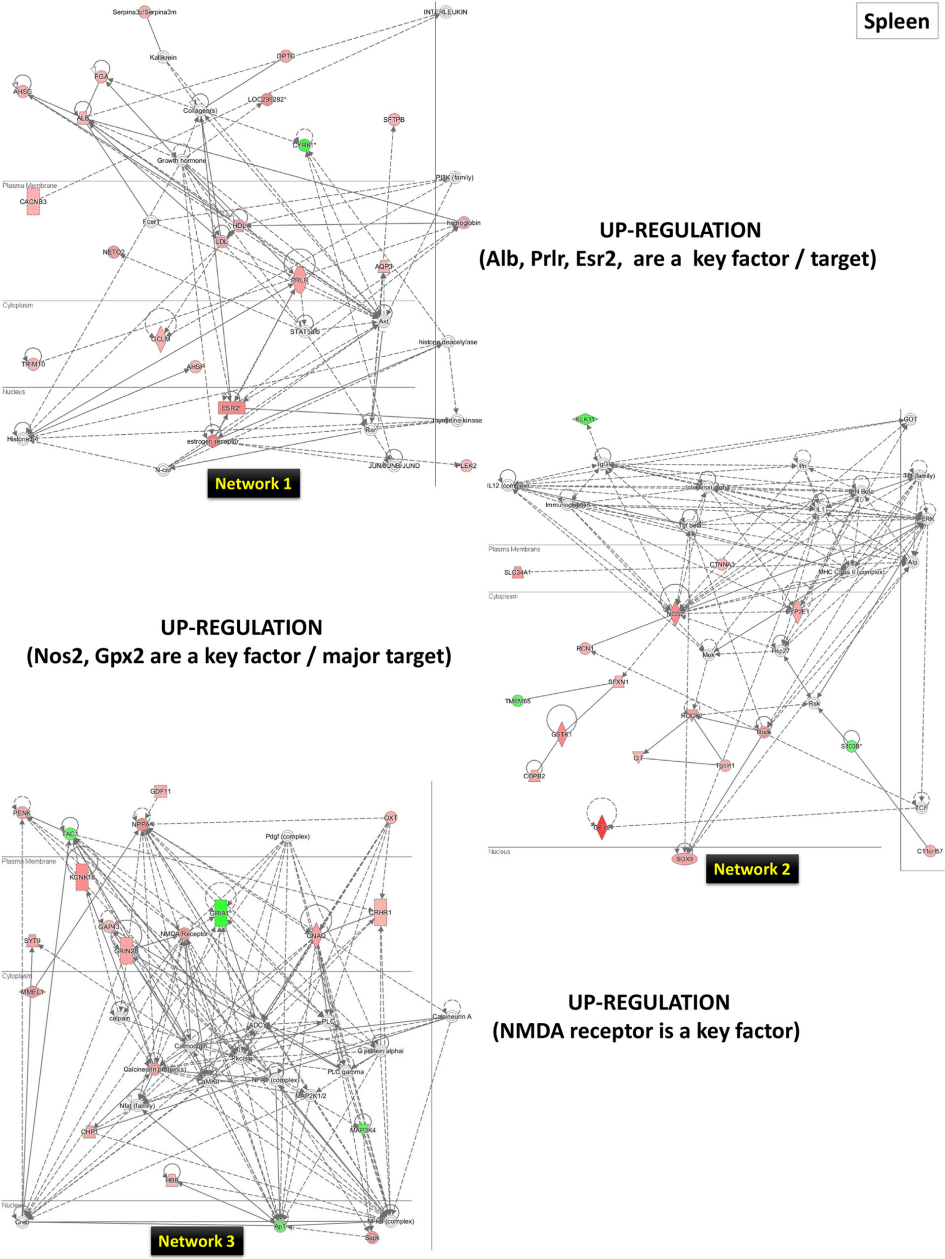
The top three networks (1, 2, and 3) for the spleen by IPA analysis. Genes central to the pathway are placed aside each network in a box. The genes (up-regulated–red; down-regulated–green) are networked based on the evidence in the IPA. For further details, see the Materials and Methods section.

**Fig 10:**
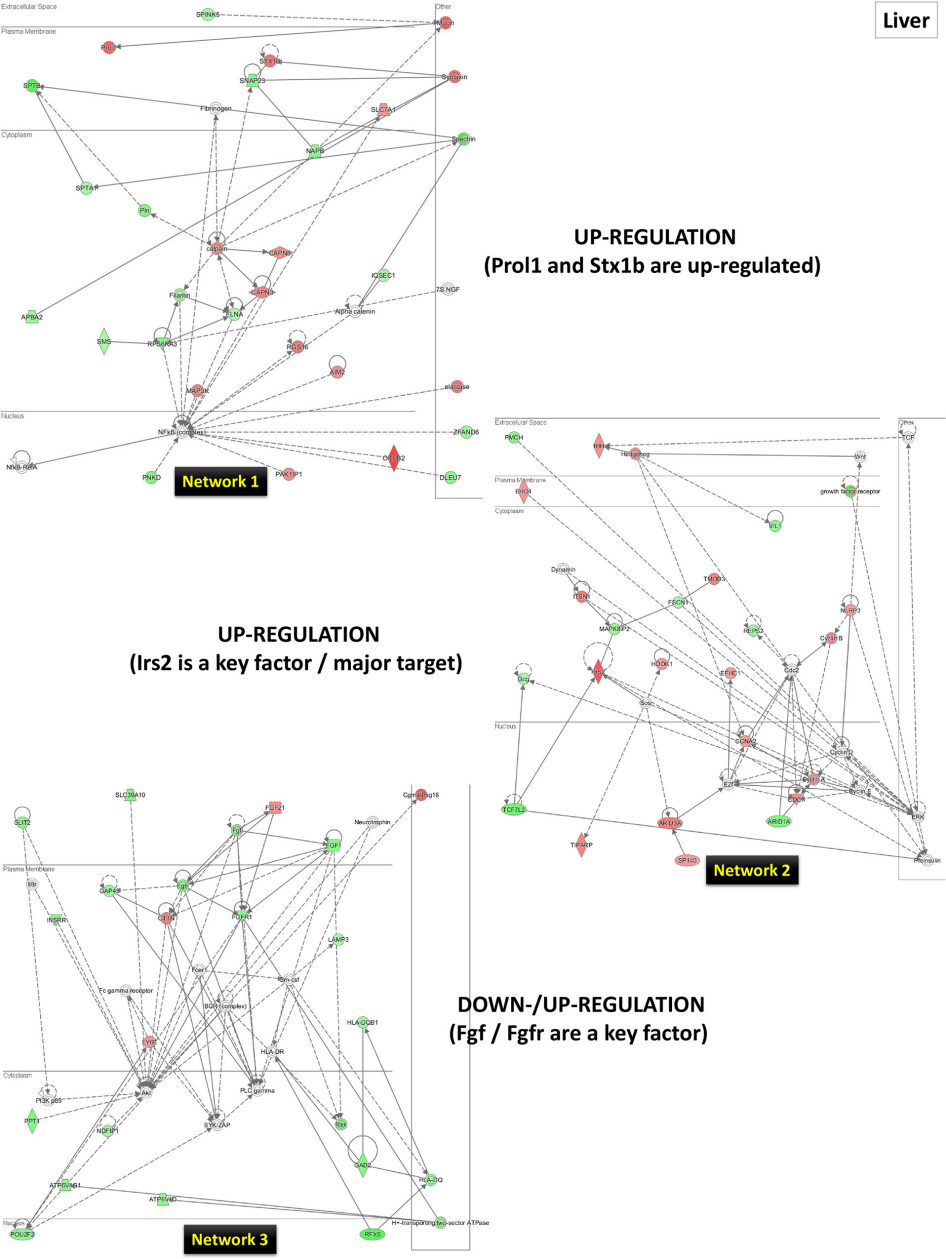
The top three networks (1, 2, and 3) for the liver by IPA analysis. Genes central to the pathway are placed aside each network in a box. The genes (up-regulated–red; down-regulated–green) are networked based on the evidence in the IPA. For further details, see the Materials and Methods section.

As a second step, for a wider bioinformatics analysis, we further performed a biological function and network analysis using the IPA tool (as described in the Materials and Methods section above). The numerous networks and pathways that were generated by this analysis revealed that LO can affect the small intestine, spleen, and the liver in complex ways. To explain the genes influenced by LO the top three networks (1, 2, and 3) have been presented and discussed in relation to the LO effects in small intestine (**Fig 8**), spleen (**Fig 9**) and liver (**Fig 10)**. Each tissue/organ IPA analysis also provided a list of top molecules that are gene candidates for the potential mechanism/s underlying the LO effects in the rat model. These genes are listed in sequence of high to low expression in **Table 2**. We further discuss these gene expression patterns and their possible role/function in the context of LO use as an aroma therapeutic treatment.

**Table 2:** The top molecules identified by IPA analysis after LO treatment.

Small intestine			Spleen			Liver		
**UP-REGULATED**								
**PAPD4**	PAP associated domain containing 4	3.09	**TRPC6**	transient receptor potential cation channel, subfamily C, member 6	4.04	**PLD2**	phospholipase D2	4.09
**OR8K5**	olfactory receptor, family 8, subfamily K, member 5	2.77	**GPX2**	glutathione peroxidase 2 (gastrointestinal)	3.86	**GIPC2**	GIPC PDZ domain containing family, member 2	2.95
**MMP19**	matrix metallopeptidase 19	2.59	**WTIP**	Wilms tumor 1 interacting protein	2.85	**RPL39L**	ribosomal protein L39-like	2.82
**RAB28**	RAB28, member RAS oncogene family	2.57	**SCAF11**	SR-related CTD-associated factor 11	2.81	**OTUB2**	OTU domain, ubiquitin aldehyde binding 2	2.72
**GADD45A**	growth arrest and DNA-damage-inducible, alpha	2.51	**IGSF8**	immunoglobulin superfamily, member 8	2.66	**NDFIP2**	Nedd4 family interacting protein 2	2.66
**ZCCHC3**	zinc finger, CCHC domain containing 3	2.49	**ACSM2A**	acyl-CoA synthetase medium-chain family member 2A	2.60	**NOS1AP**	nitric oxide synthase 1 (neuronal) adaptor protein	2.66
**PRKAR1B**	protein kinase, cAMP-dependent, regulatory, type I, beta	2.37	**LOC686142 (includes others)**	similar to Ral guanine nucleotide dissociation stimulator (RalGEF) (RalGDS)	2.55	**ONECUT3**	one cut homeobox 3	2.60
**GPRC5B**	G protein-coupled receptor, family C, group 5, member B	2.32	**ADIPOQ**	adiponectin, C1Q and collagen domain containing	2.48	**LRRC16B**	leucine rich repeat containing 16B	2.57
**CYP46A1**	cytochrome P450, family 46, subfamily A, polypeptide 1	2.29	**ESR2**	estrogen receptor 2 (ER beta)	2.36	**IL12RB2**	interleukin 12 receptor, beta 2	2.52
**FIBCD1**	fibrinogen C domain containing 1	2.27	**NOS2**	nitric oxide synthase 2, inducible	2.35	**PRRG4**	proline rich Gla (G-carboxyglutamic acid) 4 (transmembrane)	2.51
**DOWN-REGULATED**								
**SLC45A4**	solute carrier family 45, member 4	-2.63	**SLC25A23**	solute carrier family 25 (mitochondrial carrier; phosphate carrier), member 23	-2.50	**SAMT4**	spermatogenesis associated multipass transmembrane protein 4	-4.17
**ALOX12B**	arachidonate 12-lipoxygenase, 12R type	-2.63	**GRIA1**	glutamate receptor, ionotropic, AMPA 1	-2.22	**WSB1**	WD repeat and SOCS box containing 1	-3.57
**DCLK2**	doublecortin-like kinase 2	-2.38	**CYR61**	cysteine-rich, angiogenic inducer, 61	-2.17	**RHOBTB1**	Rho-related BTB domain containing 1	-3.23
**FRMD6**	FERM domain containing 6	-2.33	**KLK11**	kallikrein-related peptidase 11	-2.08	**SH3KBP1**	SH3-domain kinase binding protein 1	-3.13
**IQCK**	IQ motif containing K	-2.27	**SCD2**	stearoyl-Coenzyme A desaturase 2	-2.04	**ANKS4B**	SH3-domain kinase binding protein 1	-3.13
**PAPPA2**	pappalysin 2	-2.22	**GCG**	glucagon	-2.04	**AFF2**	AF4/FMR2 family, member 2	-2.94
**ST13**	suppression of tumorigenicity 13 (colon carcinoma) (Hsp70 interacting protein)	-2.17	**WNT5B**	wingless-type MMTV integration site family, member 5B	-1.96	**TM4SF4**	transmembrane 4 L six family member 4	-2.94
**MTMR2**	myotubularin related protein 2	-2.17	**DCLK1**	doublecortin-like Kinase 1	-1.96	**PAPLN**	papilin, proteoglycan-like sulfated glycoprotein	-2.94
**CTNNA3**	catenin (cadherin-associated protein), alpha 3	-2.13	**TAAR5**	trace amine associated receptor 5	-1.89	**ZNF280D**	zinc finger protein 280D	-2.86
**AGTR1B**	angiotensin II receptor, type 1b	-2.13	**TMEM65**	transmembrane protein 65	-1.85	**SPTB**	spectrin, beta, erythrocytic	-2.86

## Discussion

The main goal of the present study–the establishment of an animal model for investigating the effects of orally administered LO–was fulfilled by using a rat model in conjunction with an omics approach, namely DNA microarray (rat DNA microarray chip, 4×44-K) technology for the downstream analysis of target tissues/organs. The hypothesis that a daily ingestion of LO which corresponds to roughly the usual therapeutic dose in humans would cause differential gene expression at the site of absorption and metabolism, namely, the small intestine and liver, was proven by delineating the cellular responses to LO treatment. As the portal vein (portal venous system) also connects to the spleen where the blood is then transported to the liver, we also examined the spleen; moreover, it might also be a secondary target for LO. That these genome-wide changes at the target organs in this study would relate to known genes previously shown to be related to the effect of essential oils was evident from the analysis of up-regulated and down-regulated genes using both GO and biological functions analysis. In addition, many novel LO (and linalool, linalyl acetate) effects were distinguished using the IPA bioinformatics tool. We discuss below each of the six genes that were used for RT-PCR analysis and some of the top molecules and networks that were obtained from the IPA analysis in the context of LO action and probable effects.

### Gene expression analysis validation by RT-PCR and IPA analysis of top three networks 1, 2, and 3, and top molecules reveal the influence of LO treatment in a rat model

#### Highly up-regulated genes by LO treatment

*Small intestine*: *Papd4* (PAP-associated domain containing 4) gene, also known as the poly(A) RNA polymerase GLD2, encodes a protein that is involved in the process of RNA polyadenylation (http://www.ncbi.nlm.nih.gov/gene/361878). As recently reviewed, messenger RNA polyadenylation and deadenylation are critical cellular processes for the rapid regulation of gene expression under diverse conditions to which cells are exposed to [35]. Although we cannot explain the reason behind *Papd4* up-regulation by LO, one reason is obvious: the small intestinal cells were exposed to the oil-rich environment and responded by the activation of the expression of this gene. Another reason for the expression of this gene may be related to the microRNAs, their function in lipid homeostasis and interestingly where the role of especially GLD2 in the 3’-end adenylation of miR-122 was found [36,37]. Nevertheless, the possibility of LO mediating the expression of a RNA polymerase involved in microRNA stability is intriguing. This gene was also identified as a top molecule by IPA analysis, indicating its specificity to certain cellular functions that are affected by LO.

The second top-ranking molecule was a newly annotated gene *Or8k5*, which encodes for an olfactory receptor, family 8, subfamily K, member 5 protein (human: http://www.ncbi.nlm.nih.gov/gene/361878; rat ortholog: http://www.ncbi.nlm.nih.gov/gene?cmd=Retrieve&dopt=full_report&list_uids=405253). This gene is reported here for the first time to be responsible to LO treatment, and is interesting because this family of proteins as the name indicates are related to the olfactory signaling pathway and signaling by G-protein-coupled receptors (GPCR), where they function to recognize and transduce the odorant signal, in our case, LO. Although this gene function in the small intestine is a first report and its role cannot be inferred without further experimental evidence, its identification suggests that odorant receptors might also be present in the digestive tract other than the nasal tract. Interestingly, the *Or8k5* was part of the top 3^rd^ network in the small intestine further suggesting a certain role in its induction by LO (**Fig 8**, bottom network).

In addition, we also identified the *Mmp19* (matrix metallopeptidase 19) gene, which functions in calcium ion binding and metalloendopeptidase activity plays a crucial role in the pericellular proteolysis of the extracellular matrix and cell surface molecules (http://www.ncbi.nlm.nih.gov/gene/304608) as the third top-ranking molecule. Based on these known functions, the up-regulation of *Mmp19* is not unexpected, but not much is known about the physiological role of this gene in the intestine or under essential oils. In this regard, a citrus extract containing flavanones was found to induce the expression of genes that are involved in tissue repair and inflammation in human colon fibroblasts, including the *Mmp12* gene [38]. A literature survey for the *Mmp19* functions revealed two papers. In the first study, MMP19 protein was demonstrated as essential for T cell development and T cell-mediated cutaneous immune responses in mice [39]. In the second study, MMP19 protein was identified as a key regulator of lung fibrosis in mice and humans, suggesting that the up-regulation of MMP19 by lung injury may play a protective role in the development of fibrosis through the induction of prostaglandin-endoperoxide synthase 2 [40].

Another interesting gene candidate was the *Gprc5b* (GPR, family C, group 5, member B) gene, whose function is not well known but, as the name implies, has a GPCR and protein kinase activator activities (http://www.ncbi.nlm.nih.gov/gene/293546). This gene was also identified as the eight top-ranking molecules. Previously, Ito and co-workers have reported on the anatomical and histological mRNA expression profiles of orphan GPCRs in the gastrointestinal tract, and among them interestingly, the *Gprc5b* mRNA expression was found preferentially in the muscle-myenteric nerve layer, which is similar to the GPCRs expressed in central and enteric nerve systems with regulatory roles throughout the gut-brain axis [41]. A recent study reported that GPRC5B might be a control point in adipose signaling systems, linking diet-induced obesity to type-2 diabetes [42]. Is it possible that this protein might function in sensing LO and transmitting its effect via an inflammation-linked signaling pathway? Other than *Gprc5b*, the 2^nd^ top network in the small intestine revealed other GPR genes, *Gpr158*, *Gpr27*, *Gpr84*, including the chemosensory receptor family member *Taar5* (trace amine-associated receptor) further suggesting a certain role for the induced expression of these transmembrane receptors by LO in sensing the volatiles in its induction by LO (**Fig 8**, center network). In particular, a recent report on the human TAAR5 revealed its specific activation by a highly volatile aminic compound, trimethylamine, indicating its role as a molecular sensor for detecting volatile amines [43]. In our study, identification of *Taar5* gene expression and the generated network (top network 2) revealing a cluster of GPRs and TAAR5 suggests the first step in sensing LO might take place in the small intestine.

*Spleen*: In the spleen, we were not able to identify a major factor *per se*, but the three generated networks gave a hint into the molecular events being modulated by LO treatment. In the top-ranking network 1, some of the key molecules were albumin (*Alb*), prolactin receptor (*Prlr*), and estrogen receptor 2 beta (*Esr2*). The *Alb* (albumin, a plasma protein) gene is associated with numerous binding functions and processes and serves a stress biomarker (http://www.ncbi.nlm.nih.gov/gene/24186). Does LO induce *Alb* gene expression as a stress effect of the oil? Although the extrahepatic synthesis of plasma proteins in mammalian tissues has been reported during stress/inflammation [44], it is not known what role the spleen plays in albumin synthesis. Recently and again in the liver, it was shown that the immunomodulatory and antioxidant function of albumin stabilizes the endothelium and improves survival in a rodent model of chronic liver failure [45]. Thus, *Alb* expression might be a positive reaction to the perception of LO in the intestine; however, it would be necessary to check the levels of serum/plasma albumin in future studies. In network 1 (**Fig 9**, upper network), we found a link to both *Alb* and AHSG (alpha-2-HS-glycoprotein), which is quite interesting because this protein has been recently linked to obstructive sleep apnea patients with cardiovascular diseases as a salivary biomarker [46]. It is tempting to link this finding to the use of LO in helping people relax and sleep [47–49].

The *Prlr* gene (http://www.ncbi.nlm.nih.gov/gene/24684) was found to interact with the *Esr2* (http://www.ncbi.nlm.nih.gov/gene/25149) gene. Although the link to LO is not known, prolactin, a pituitary hormone well known for its role in reproduction also influences the immune system via binding to its receptor (PRLR) in the immune cells [50]. Looking at the top network 1, some key genes that are well known to be involved downstream of the activation of the prolactin receptor also grouped in the pathway, namely signal transducer and activator of transcription (*Stat5a/b*) and protein kinase B (*Akt*); though these gene expressions were not at the cut-off range in our DNA microarray analysis. Both the STAT and AKT pathways are also important molecules/signaling points downstream of the cellular signals, including growth factors, cytokines, etc [51–53], suggesting that LO (linalool/linalyl acetate) might act as a potent cellular signal. Another interesting gene that was identified as the ninth ranking molecule and in network 1 as the central molecule was the *Esr2* gene that binds estrogen and mediates transcriptional activation (http://www.ncbi.nlm.nih.gov/gene/25149). The *Esr2* gene was a surprising discovery, and subsequent studies have established that this ERbeta as it is also known plays only a minor role in mediating estrogen action in uterus and other classical estrogen target tissues but has a more defined role in the ovary, cardiovascular system and brain, including animal models of inflammation [54]. However, there is a demonstrated link between prolactin and stimulation of ERalpha and ERbeta in rat corpus luteum and decidua of pregnancy [55]. Interestingly, mutation studies of the *Stat5* response elements indicated that PRL regulation of ER expression requires both intact Stat5 binding sites as well as functional Stat5 [55].

The *Trpc6* (transient receptor potential cation channel, subfamily C, member 6) gene (http://www.ncbi.nlm.nih.gov/gene/7225) encoding non-selective cation channels [56,57] was also identified as a top molecule by IPA analysis (it was also a key molecule in network 6; data not shown). Much is known about this gene in the kidney functions and its diseases [58], and *Trpc6* function has been identified in smooth muscle and axonal guidance. Although we know very little about its role in the intestine, LO becomes a new extracellular stimuli triggering its activation. Because the TRPC6 protein stimulates calcium influx [59,60] via phospholipase C-mediated signals [58] and through angiotensin II activation involving reactive oxygen species (ROS) in rat podocytes [61], it is possible that LO triggers some type of signaling cascade activating these channels directly or indirectly. Another recent study targeting the brain linked oral LO use in patients suffering from subsyndromal anxiety and the involvement of voltage-dependent calcium channels [62]. Regarding ROS, two key molecules were mapped in the second-ranking network 2, namely, nitric oxide (NO) synthase 2 (*Nos2*, inducible; *iNos*) and glutathione peroxidase family, the glutathione peroxidase 2 (*Gpx2*) genes. *Nos2* is a cytokine-inducible enzyme involved in NO production (http://www.ncbi.nlm.nih.gov/gene/24599) and was identified as the 10^th^ top-ranking molecule. Though there is no study available on LO causing the induction of *Nos2* gene expression or activity, two reports exist in the literature showing iNOS protein expression (and NO production) by oleuropein (bitter principle of olives) in mouse macrophages [63] and in lung tissues of a murine model following *Nigella sativa* fixed oil and dexamethasone treatment [64]. *Gpx2* is involved in the negative regulation of the inflammatory response to antigenic stimulus (http://www.ncbi.nlm.nih.gov/gene/29326) as the second top-ranking molecule. *Gpx2* gene knockout studies in mice showed that the mRNA expression of *Gpx2* and GPX-2 activity levels are induced by luminal microflora and demonstrated a role for GPX as an anti-inflammatory molecule [65]. Moreover, this gene was also identified as playing a role in alleviating the apoptotic response of MCF7 cells to oxidative stress [66].

In the third top-ranking network, the N-methyl-D-aspartate (NMDA) receptor appears as a key molecule. The beneficial effects of LO has been thought to be mediated in part through interaction with the NMDA or GABA_A_ receptors, voltage dependent sodium channels or cholinergic neurotransmission ([62,67], and references therein). This may be the first report showing that NMDA receptor mRNA expression in the spleen can be increased by LO treatment.

*Liver*: The *Cyp2c12* (cytochrome P450, family 2, subfamily c, member 12; coding for steroid hydroxylases) gene is involved in oxidation-reduction processes (http://www.ncbi.nlm.nih.gov/gene/25011). Although we cannot explain the reason for up-regulation of this liver enzyme gene, it is interesting to note that there is a gender-related regulation of this gene expression in rats [68], with modulation by growth hormone (GH) [69] and under cholestasis [70]. Many questions remain than are answered by this gene induction by LO, primarily whether LO is being simply metabolized by this enzyme or if there is more to the effects of LO in the context of sexual dimorphism and GH effects. The *Pld2* (phospholipase D2) gene encodes an enzyme that catalyzes the hydrolysis of phosphatidylcholine to phosphatidic acid and choline and is involved therein in signal transduction, membrane trafficking and mitosis regulation (http://www.ncbi.nlm.nih.gov/gene/25097). This gene was also identified as a top molecule by IPA analysis. In general, the action of phospholipases, including PLD and PLC, produces lipid mediators, including prostaglandins and metabolites of arachidonic acid, which play important roles in hepatocyte proliferation. We also identified the one cut homeobox 3 (*Onecut3*) gene encoding for the ONECUT class of transcription factors. The ONECUT1 (HNF-6) is the main member which was shown to be involved in endoderm differentiation to various organs, including the pancreas and liver, including working as part of a regulatory cascade between two paralogous transcription factors, OC-1 and OC-3 [71,72]. Recently, HNF-6, OC-2 and OC-3 (*Onecut3*) have been found to also control the development of Locus Coeruleus (the main noradrenenergic nucleus in the vertebrate central nervous system), which regulates several processes such as arousal, sleep, adaptive behaviors and stress [73]. We cannot explain the presence of *Onecut3* gene and its induction by LO treatment in the liver, at present.

Looking at networks 1 to 3, we could not find some key molecules or factors but individual genes up-regulated / down-regulated in each network. We further discuss some of these highly changed molecules. The network 1 contained a highly induced gene, *Prol1* (proline rich, lacrimal 1). There is no literature on the *Prol1* gene induction by essential oils, but the human opiorphin, a recently identified endogenous pentapeptide encoded by *Prol1*, was shown to contribute to cardiovascular modulation / blood pressure through the angiotensin II pathway [74]. In addition, calpain proteins, encoded by *Capn8* (http://www.ncbi.nlm.nih.gov/gene/170808) and *Capn3* (http://www.ncbi.nlm.nih.gov/gene/29155) genes were prominently present in the network 1. The calcium-dependent proteases have numerous functions including cell survival, plasticity, and motility, though we do not anything about their function in liver following LO treatment. In network 2, insulin receptor substrate 2 (*Irs2*) gene was a main molecule, and its encoded protein plays a role in insulin-stimulated fetal liver growth (http://www.ncbi.nlm.nih.gov/gene/29376). The IRS2, an essential insulin receptor adaptor protein was shown to be involved in the regulation of hepatic lipid metabolism by adiponectin via IRS-2 phosphorylation in OLETF rats [75], and most recently a critical role in hepatic insulin signaling via the liver Hif-2α-Irs2 pathway that is modulated by Vegf inhibition [76]. In the network 3, fibroblast growth factor (FGF) was the dominant molecule, with *Fgf1* (http://www.ncbi.nlm.nih.gov/gene/25317), Fgf21 (http://www.ncbi.nlm.nih.gov/gene/170580), and *Fgfr1* (http://www.ncbi.nlm.nih.gov/gene/79114) genes being differentially expressed. In the liver, FGFR signaling has been demonstrated to play a central role in repair after liver injury, i.e., it is crucial for liver homeostasis and regeneration [77]. Most recently, a breakthrough has been made in the use of FGF1 as a therapeutic agent for the treatment of insulin resistance and type-2 diabetes [78]. In case of the *Fgf21*, a role for this factor in metabolic adaptations, not only in the ketotic sate but also to face an unbalanced nutritional situation was suggested [79]. A recently published report has provided novel insight into FGF21-induced cardiac effects in obesity and ischemia [80].

### Down-regulated genes by LO treatment

*Small intestine*: Although there is no information on the *Lrp1b* (low-density lipoprotein receptor-related protein 1B) gene (http://www.ncbi.nlm.nih.gov/gene/311926) in relation to the intestines, a recent report indicated that LRP1B protein may function as a tumor suppressor against renal cell cancer (RCC) and is possibly be involved in the regulation of cell motility and actin cytoskeleton reorganization in RCC [81]. In addition, in network 1, the *Tnf* (tumor necrosis factor) gene, which acts as a cytokine and binds TNF receptors, playing a role in the induction of apoptosis and inflammatory response (http://www.ncbi.nlm.nih.gov/gene/?term=NM_012675), was also identified as the central molecule. LO has proven benefits for inflammatory bowel disease along with reduced levels of TNF-a, strongly supporting a potential for LO in maintaining intestinal health by modulating the enteric microbial as recently demonstrated by the use of *Lavandula* × intermedia cultivar Okanagan lavender (OLEO) in a mouse model of acute colitis caused by *Citrobacter rodentium* [82]. We also identified two newly annotated down-regulated top molecules. The first was the *Iqck* (IQ motif containing K) gene, where the IQ motif serves to bind EF-hand proteins such as calmodulin (http://www.ncbi.nlm.nih.gov/gene/100909537). Its function in rat remains unknown, but a human homolog was (http://www.ncbi.nlm.nih.gov/gene/124152) was identified as a potential candidate gene for obsessive-compulsive disorder in a genome-wide association study [83]. The other was *Pappa2* (pappalysin2; pregnancy-associated plasma protein-A2) gene which encodes for a member of the pappalysin family of metzincin metalloproteinases (http://www.ncbi.nlm.nih.gov/gene/364027). PAPPA and PAPPA2 proteins are highly expressed in the human placenta, and known to cleave the insulin-like growth factor (IGF)-binding proteins thereby increasing the availability of growth factors [84]. PAPPA is well recognized as a marker for fetal genetic disorders and adverse pregnancy outcomes, however, there physiological role of PAPPA2 remains unclear. A recent study showed PAPPA2 up-regulation in severe pre-eclampsia and, functionally, this may be mediated via increased placental hypoxia known to occur with this pregnancy disorder [85]. How these proteins relate to the effect of LO remains to be uncovered, but a hint may lie in a previous report where genetic down-regulation of PAPPA by a protease inhibitor reduced ovarian cancer cell growth, invasion and metastatis via suppression of IGF-1-dependent Akt and ERK1/2 activation [86].

*Spleen*: The Cyr61 (cysteine-rich angiogenic inducer 61) gene encodes an extracellular binding protein (http://www.ncbi.nlm.nih.gov/gene/83476). Numerous experiments have revealed that Cyr61 plays diverse roles in cellular proliferation, migration, differentiation, and angiogenesis, biological phenomena that are required for tumor growth and metastasis [87]. How LO affects this gene expression and for what purpose remain to be identified. This gene was also identified as the third top molecule by IPA analysis. The Slc25a23 (solute carrier family 25, mitochondrial carrier/phosphate carrier, member 23) gene (http://www.ncbi.nlm.nih.gov/gene/301113) was also identified as a top molecule by IPA analysis, though its function remains to be clarified. One newly annotated down-regulated molecule was the Dclk1 (doublecortin-like kinase 1; http://www.ncbi.nlm.nih.gov/gene/83825) gene, however its function also remains to be clarified in context of LO treatment. Interestingly, DCLK1 is considered as a putative pancreatic stem cell marker and is up-regulated in pancreatic cancer, colorectal cancer and many other solid tumors [88], and might be a candidate for developing chemotherapeutic agents.

*Liver*: The down-regulation of the Cxcl1 (chemokine (C-X- motif) ligand 1) gene (http://www.ncbi.nlm.nih.gov/gene/81503) was observed with LO. A literature survey quickly revealed that CXCL1 down-regulation inhibits tumor growth in colorectal liver metastasis [89]. The Samt4 (spermatogenesis-associated multipass transmembrane protein 4) gene (http://www.ncbi.nlm.nih.gov/gene/685774) was identified as a top molecule by IPA analysis, but we are at loss to explain this gene expression. In addition, in network 1, the Ccnb1 (cyclin B1) gene (http://www.ncbi.nlm.nih.gov/gene/25203) was also identified as the central molecule, although here also we could not assign its role in the context of LO action. On the other hand, four newly annotated down-regulated molecules were identified, namely the Aff2 (AF4/FMR2 family, member 2;http://www.ncbi.nlm.nih.gov/gene/293922), Papln (papilin, proteoglycan-like sulfated glycoprotein; http://www.ncbi.nlm.nih.gov/gene/314297), Znf280d (zinc finger protein 280D; http://www.ncbi.nlm.nih.gov/gene/315798), and Sptb (spectrin, beta, erythrocytic; http://www.ncbi.nlm.nih.gov/gene/314251) genes. Functions for these newly annotated genes remain unclear. However, for the Aff2, its human ortholog, variously called FMR2 / MRX2 / OX19 / FMR2P / FRAXE is associated with the fragile X E syndrome, a form of nonsyndromic X-linked mental retardation (http://www.ncbi.nlm.nih.gov/gene/2334).

## Conclusions

We potentially identified some of the LO influenced genes that might be involved in mediating the beneficial effects of this essential oil as an aromatherapy agent. The gene inventory for the three tissues/organs, small intestine, spleen, and liver serves as a valuable resource for further analysis and study. Based on the above identified differentially expressed genes by DNA microarray analysis and subsequent bioinformatic analyses, the essential oil of lavender (LO) can cause a moderate activation of the small intestine, spleen, and liver inflammation- and lipid homeostasis-related functions among other functions that are related to innate immunity and the immune system. To note, LO may not be toxic (to rats) at the dosage used in this study as on previous research evaluating the developmental toxicity of linalool in rats has indicated that the maternal “no observed adverse effect level (NOAEL) was 500 mg/kg/day compared to the developmental NOAEL at 100 mg/kg/day [90]. These gene inventories are the first step in future experiments that will be required to reveal their precise function to confirm the recognized effects of LO on humans. These experiments may involve checking, for example, the effects of LO on stress or depression and sleep disorder models of animals to confirm the involvement of some of the potential gene candidates that were screened in the present study. Further functional analysis will be required to characterize the role of the genes that were identified in this study, and only then will it be possible to facilitate a deeper understanding on how aroma oil benefits the human body. On the other, there are some limitations to our study. The first order of research would be an exhaustive bioinformatics analysis and interplay of the molecular factors at least among these three tissues/organs examined in the current study by extensively utilizing all available functions in the IPA tool. The other immediate priority would be to analyze the missing link in these transcriptomic data, namely the DNA microarray analysis of the blood (raw transcriptomic data has just been submitted at the GEO website under public access; accession number-GSE67499). The reasons are two-fold. First, blood is transporting the LO (linalool and linalyl acetate) through the portal vein, and second, as at least linalool was present at higher levels after LO treatment the blood should show transcriptomal information/changes and which might also be a valuable resource in pharmagenomic studies vis-à-vis LO. As also rightly stated by the anonymous referee, the appearance of linalool and linalyl acetate (and possibly other LO components) in the portal venous sample but not in venous (systemic) blood adds a level of complexity to interpret different gene expressions in different tissues relative to the hepatic-portal versus systemic circulation. Understanding this would be essential to predict or test responses in other tissues downstream from this hepatic-portal system, especially in the context of the human use of LO for so many different effects. One such target is the brain, as our research group is interested in the effects of molecules/peptides and natural (biologically active) compounds on the brain, vis-à-vis neuroprotection.

## Supporting Information

S1 Fig Plasma linalool and linalyl acetate concentration in the portal vein after oral administration of lavender oil (LO). LO was administrated to male SD rats (n = 3) at a dose of 1.25 mg/kg. Blood samples (5 mL) were collected, in blood collection tubes containing 3.2% sodium citrate (TERUMO Corporation, Tokyo, Japan), from the portal vein 5, 10, 15, 30, and 60 min after oral administration of LO. The plasma was centrifuged (3000 rpm, 10 min, 4°C) and the supernatant was stored at -80°C. The metabolites were extracted using a Bond-Elut-C18 resin column (100 mg/1 mL). Determination of the two metabolites linalool and linalyl acetate (standards were obtained from Wako Pure Chemical Industries Pvt. Ltd., Osaka, Japan) was carried out using a SHIMADZU GC-MS QP2010plus (Kyoto, Japan) and a Rtx-5MS column (30 m x 0.25 mm i.d., 0.25 mm d.f.; RESTEK, Bellefonte, PA, USA). Conditions; interface heating: 250°C; temperature program: 60°C (1 min) – 200°C (1 min)- 250°C (1 min); injected volume: 1 mL; split-ratio: 50.0; carrier gas: helium. Discussion is in the text.
(PPTX)

S2 Fig The detailed protocol for total RNA extraction from rat small intestine, spleen, and liver. (PPTX)

S3 Fig Expression level of the *Gapdh* gene, by expressed level of probe signal intensity in Cy3 and Cy5 labels under DNA microarray experiment in the rat small intestine (A), spleen (B), and liver (C). Ten probes for the *Gapdh* gene are plotted for both labels, Cy3 and Cy5. DNA microarray was performed as described in Materials and Methods section.
(PPTX)

S1 Table Lavender oil small intestine up- and down-regulated genes. (XLSX)

S2 Table Lavender oil spleen up- and down-regulated genes. (XLSX)

S3 Table Lavender oil liver up- and down-regulated genes. (XLSX)

S4 Table Function of rat small intestine genes up-regulated by LO. (XLSX)

S5 Table Function of rat small intestine genes down-regulated by LO. (XLSX)

S6 Table Function of rat spleen genes up-regulated by LO. (XLSX)

S7 Table Function of rat spleen genes down-regulated by LO. (XLSX)

S8 Table Function of rat liver genes up-regulated by LO. (XLSX)

S9 Table Function of rat liver genes down-regulated by LO. (XLSX)
