# Supplementary material for: Unraveling the Rat Intestine, Spleen and Liver Genome-Wide Transcriptome after the Oral Administration of Lavender Oil by a Two-Color Dye-Swap DNA Microarray Approach
Source: PLoS One. 2015 Jul 10;10(7):e0129951. doi: 10.1371/journal.pone.0129951 (PMC4498626; doi:10.1371/journal.pone.0129951)
Supplement: S1 Fig — LO was administrated to male SD rats (n = 3) at a dose of 1.25 mg/kg. Blood samples (5 mL) were collected, in blood collection tubes containing 3.2% sodium citrate (TERUMO Corporation, Tokyo, Japan), from the portal vein 5, 10, 15, 30, and 60 min after oral administration of LO. The plasma was centrifuged (3000 rpm, 10 min, 4°C) and the supernatant was stored at -80°C. The metabolites were extracted using a Bond-Elut-C18 resin column (100 mg/1 mL). Determination of the two metabolites linalool and linalyl acetate (standards were obtained from Wako Pure Chemical Industries Pvt. Ltd., Osaka, Japan) was carried out using a SHIMADZU GC-MS QP2010plus (Kyoto, Japan) and a Rtx-5MS column (30 m x 0.25 mm i.d., 0.25 mm d.f.; RESTEK, Bellefonte, PA, USA). Conditions; interface heating: 250°C; temperature program: 60°C (1 min) – 200°C (1 min)- 250°C (1 min); injected volume: 1 mL; split-ratio: 50.0; carrier gas: helium. Discussion is in the text. (PPTX) [file pone.0129951.s001.pptx]

## Slide 1
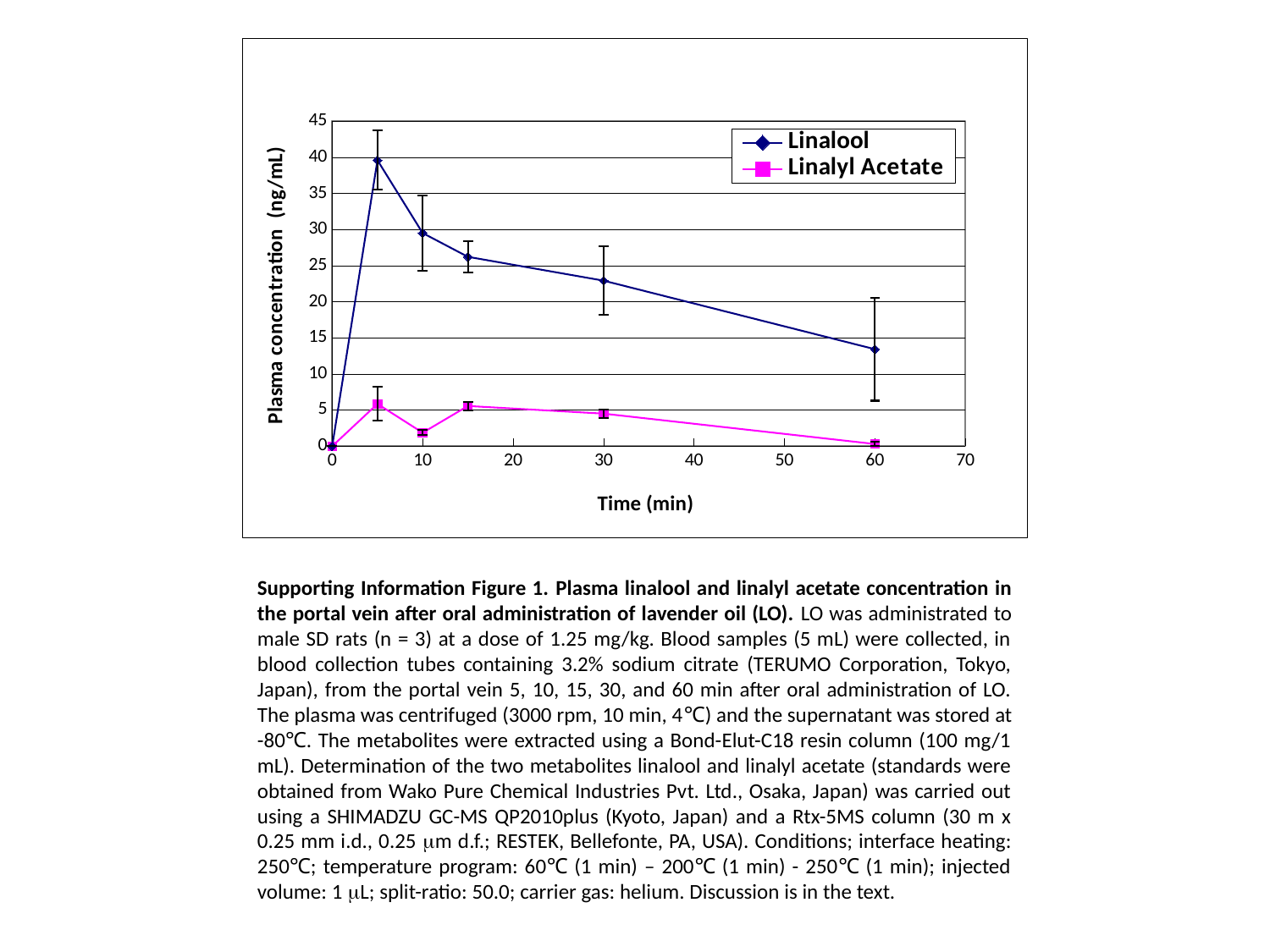

### Chart
| Category | Linalool | Linalyl Acetate |
|---|---|---|Supporting Information Figure 1. Plasma linalool and linalyl acetate concentration in the portal vein after oral administration of lavender oil (LO). LO was administrated to male SD rats (n = 3) at a dose of 1.25 mg/kg. Blood samples (5 mL) were collected, in blood collection tubes containing 3.2% sodium citrate (TERUMO Corporation, Tokyo, Japan), from the portal vein 5, 10, 15, 30, and 60 min after oral administration of LO. The plasma was centrifuged (3000 rpm, 10 min, 4℃) and the supernatant was stored at -80℃. The metabolites were extracted using a Bond-Elut-C18 resin column (100 mg/1 mL). Determination of the two metabolites linalool and linalyl acetate (standards were obtained from Wako Pure Chemical Industries Pvt. Ltd., Osaka, Japan) was carried out using a SHIMADZU GC-MS QP2010plus (Kyoto, Japan) and a Rtx-5MS column (30 m x 0.25 mm i.d., 0.25 mm d.f.; RESTEK, Bellefonte, PA, USA). Conditions; interface heating: 250℃; temperature program: 60℃ (1 min) – 200℃ (1 min) - 250℃ (1 min); injected volume: 1 mL; split-ratio: 50.0; carrier gas: helium. Discussion is in the text.
