# Supplementary material for: Unraveling the Rat Intestine, Spleen and Liver Genome-Wide Transcriptome after the Oral Administration of Lavender Oil by a Two-Color Dye-Swap DNA Microarray Approach
Source: PLoS One. 2015 Jul 10;10(7):e0129951. doi: 10.1371/journal.pone.0129951 (PMC4498626; doi:10.1371/journal.pone.0129951)
Supplement: S2 Fig — (PPTX) [file pone.0129951.s002.pptx]

## Slide 1
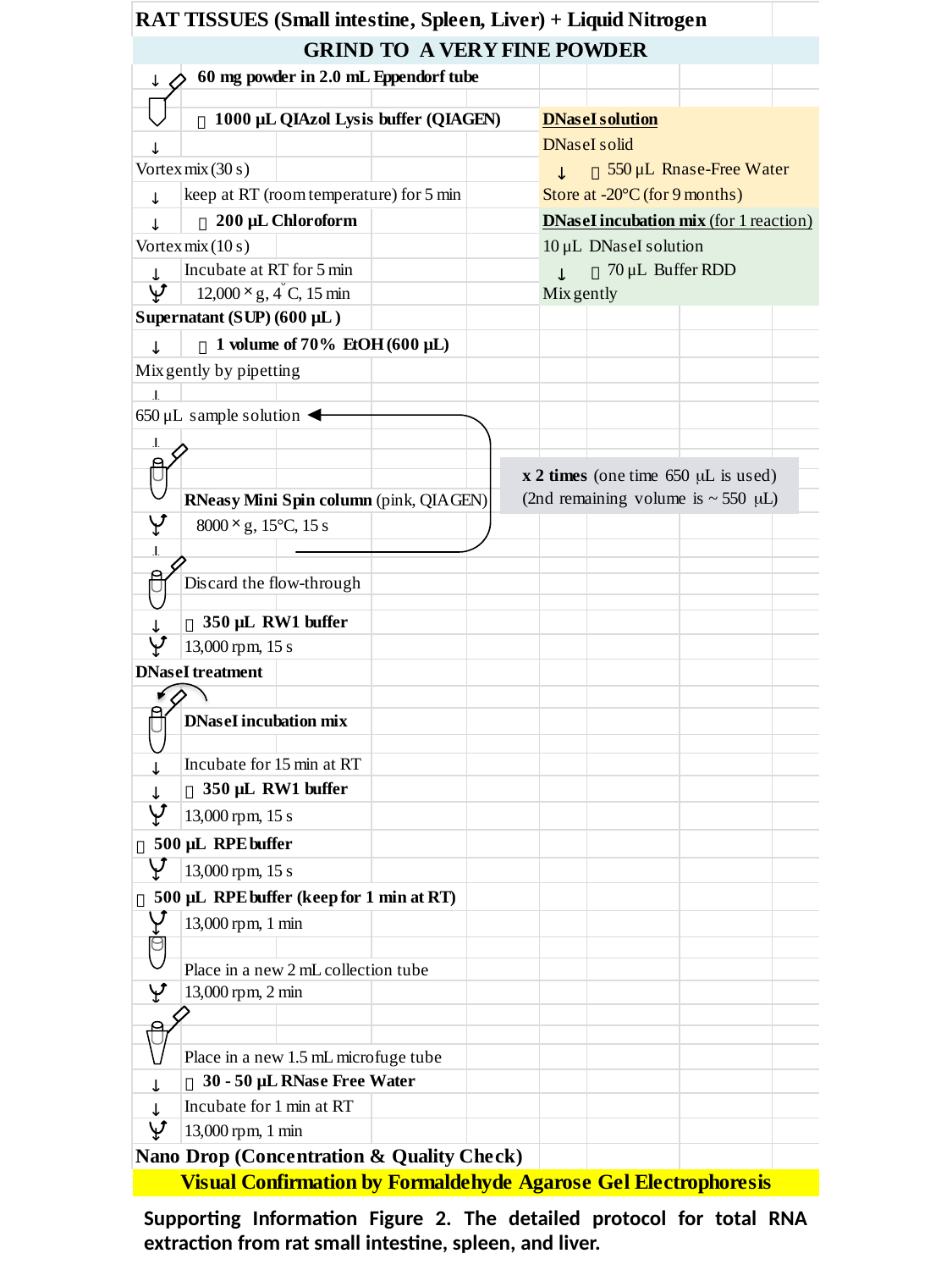

Supporting Information Figure 2. The detailed protocol for total RNA extraction from rat small intestine, spleen, and liver.
