# Supplementary material for: Unraveling the Rat Intestine, Spleen and Liver Genome-Wide Transcriptome after the Oral Administration of Lavender Oil by a Two-Color Dye-Swap DNA Microarray Approach
Source: PLoS One. 2015 Jul 10;10(7):e0129951. doi: 10.1371/journal.pone.0129951 (PMC4498626; doi:10.1371/journal.pone.0129951)
Supplement: S3 Fig — Ten probes for the Gapdh gene are plotted for both labels, Cy3 and Cy5. DNA microarray was performed as described in Materials and Methods section. (PPTX) [file pone.0129951.s003.pptx]

## Slide 1
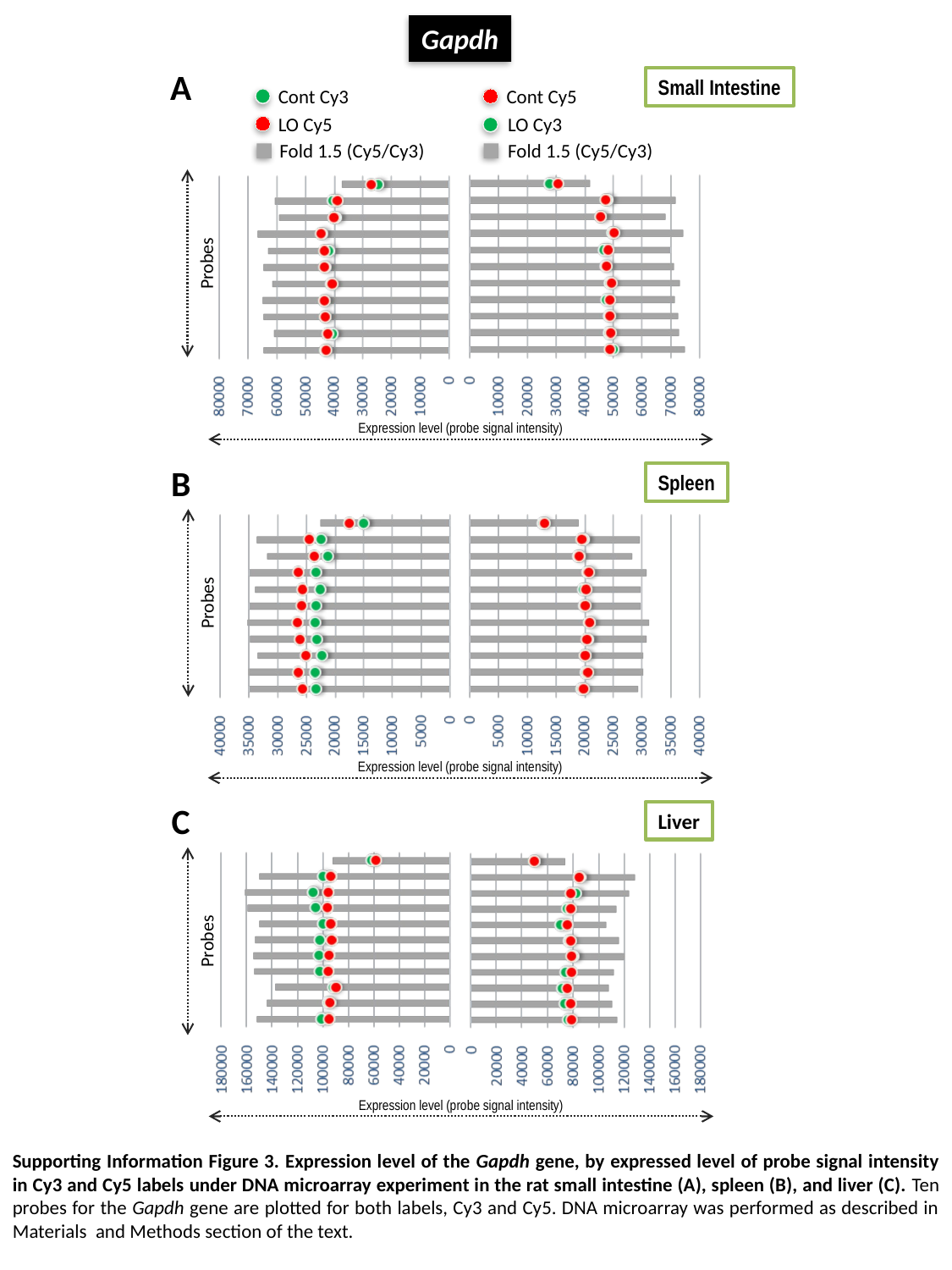

Gapdh
A
Small Intestine
Cont Cy3
LO Cy5
Fold 1.5 (Cy5/Cy3)
Cont Cy5
LO Cy3
Fold 1.5 (Cy5/Cy3)
Probes
Expression level (probe signal intensity)
B
Spleen
Probes
Expression level (probe signal intensity)
C
Liver
Probes
Expression level (probe signal intensity)
Supporting Information Figure 3. Expression level of the Gapdh gene, by expressed level of probe signal intensity in Cy3 and Cy5 labels under DNA microarray experiment in the rat small intestine (A), spleen (B), and liver (C). Ten probes for the Gapdh gene are plotted for both labels, Cy3 and Cy5. DNA microarray was performed as described in Materials and Methods section of the text.
